# Modulatory role of radioprotective 105 in mitigating oxidative stress and ferroptosis via the HO-1/SLC7A11/GPX4 axis in sepsis-mediated renal injury

**DOI:** 10.1038/s41420-025-02578-7

**Published:** 2025-07-01

**Authors:** Hong Duo, Yanwei Yang, Jun Luo, Yumeng Cao, Qian Liu, Jiarui Zhang, Siqi Du, Jian You, Guqin Zhang, Qifa Ye, Huaqin Pan

**Affiliations:** 1 National Quality Control Center for Donated Organ Procurement, Hubei Key Laboratory of Medical Technology on Transplantation, Hubei Clinical Research Center for Natural Polymer Biological Liver, Hubei Engineering Center of Natural Polymer-Based Medical Materials, Zhongnan Hospital of Wuhan University, Institute of Hepatobiliary Diseases of Wuhan University, Transplant Center of Wuhan University, Wuhan, Hubei China; 2 Department of Critical Care Medicine, Zhongnan Hospital of Wuhan University, Clinical Research Center of Hubei Critical Care Medicine, Wuhan, 430071 China; 3 Nanchang Hongdu Hospital of Traditional Chinese Medicine, 330006 Jiangxi, China; 4 College of Life Sciences, Wuhan University, Wuhan, 430072 Hubei China; 5 Department of Respiratory and Critical Care Medicine, Zhongnan Hospital of Wuhan University, Wuhan, 430071 China; 6 Zhongnan Hospital of Wuhan University, Institute of Hepatobiliary Diseases of Wuhan University, Transplantation Intensive Care Unit, Transplant Center of Wuhan University, Hubei Key Laboratory of Medical Technology on Transplantation, Wuhan, 430071, China; Department of Critical Care Medicine, Zhongnan Hospital of Wuhan University, Clinical Research Center of Hubei Critical Care Medicine, Wuhan, 430071 China

**Keywords:** Immune cell death, Cell death, Acute kidney injury

## Abstract

Sepsis-associated acute kidney injury (SA-AKI) is a critical condition characterized by high morbidity and mortality rates, particularly in intensive care settings. This study focuses on RP105, a pattern recognition receptor, exploring its role in moderating the mechanisms of oxidative stress and ferroptosis during SA-AKI, offering insights into its potential as a therapeutic target. SA-AKI model was established using RP105 knockout (KO) and wild-type (WT) mice through cecal ligation and puncture (CLP). Comprehensive evaluations included the assessment of ferroptosis markers and the expression levels of pro-inflammatory cytokines. RP105 expression was markedly reduced in the kidneys following CLP induction, correlating with worsened renal outcomes. Compared to the Sham group, RP105^−/−^ mice displayed heightened renal damage, increased levels of oxidative stress markers, and enhanced lipid peroxidation. Notably, the deficiency of RP105 led to increased macrophage infiltration and a shift towards pro-inflammatory phenotypes, which further potentiated ferroptosis and exacerbated renal tissue damage. By influencing macrophage behavior and mitigating inflammatory responses. RP105 deficiency exacerbates macrophage-induced inflammation, oxidative stress, and ferroptosis, forming a vicious cycle that leads to more severe renal injury. These findings underscore the pivotal role of RP105 in mitigating oxidative stress and suppressing ferroptosis in the context of SA-AKI through regulation of the HO-1/SLC7A11/GPX4 axis. By preventing macrophage polarization toward a pro-inflammatory phenotype, RP105 alleviates inflammatory responses and tissue damage, highlighting its potential as a therapeutic target. Thus, RP105 emerges as a promising therapeutic candidate for mitigating sepsis-induced renal damage.

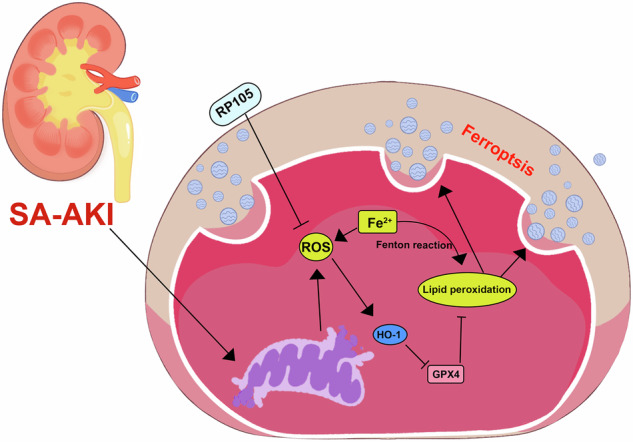

## Introduction

Acute kidney injury (AKI) is a complex disorder associated with systemic inflammatory responses, characterized by the acute loss of kidney function, leading to electrolyte imbalances, fluid dysregulation, and the accumulation of metabolic waste products [1, 2]. AKI is common among critically ill patients, with sepsis-associated acute kidney injury (SA-AKI) being one of the leading causes, carrying a mortality rate of 50–70%, and posing a significant global health challenge [3, 4]. Sepsis is defined as a life-threatening organ dysfunction caused by a dysregulated host response to infection, often described as a “failed starvation response,” reflecting severe metabolic stress with the production of high-energy metabolites such as lactate and free fatty acids [5]. Although the exact pathogenesis of SA-AKI remains incompletely understood, current research suggests it involves processes such as ferroptosis, lipid peroxidation, ischemia-reperfusion injury, immune dysregulation, systemic inflammation, oxidative stress, autophagy, microcirculatory dysfunction, and tubular damage [6–10]. Investigating these mechanisms may provide new insights for the management and treatment of sepsis.

The onset of sepsis is typically initiated by pathogen invasion (e.g., bacteria or viruses), triggering a host immune response. Innate immune cells, such as macrophages, monocytes, and neutrophils, recognize pathogen-associated molecular patterns (PAMPs), such as bacterial lipopolysaccharides (LPS), via pattern recognition receptors like Toll-like receptors (TLRs) [11–13]. A hallmark of sepsis is the dysregulated immune response, where excessive inflammation causes significant tissue damage and oxidative stress, particularly affecting renal cells [14–16]. Disturbances in iron metabolism play a critical role, leading to an expansion of unstable intracellular iron pools. Excess divalent iron catalyzes the generation of lipid peroxides, damaging cellular organelles. Notably, bacterial proliferation depends on iron availability, and ferroptosis, an iron-dependent form of cell death, releases excess intracellular iron, facilitating bacterial growth [17–19]. This, in turn, promotes lipid peroxidation through the release of fatty acids and ROS, creating a vicious cycle that exacerbates infection and ultimately results in SA-AKI [5, 20–22].

Ferroptosis, as a newly identified form of programmed cell death, is distinct from other types of cell death in morphology, biology, and genetics. It is characterized by iron metabolism-mediated lipid peroxidation and glutathione depletion, leading to redox imbalance [10, 23, 24]. Through the Fenton reaction, iron interacts with polyunsaturated fatty acids (PUFAs) to generate lipid hydroperoxides (LOOHs), which accumulate when antioxidant systems fail. This accumulation disrupts glutathione (GSH) synthesis, leading to the inactivation of glutathione peroxidase 4 (GPX4), ultimately resulting in the accumulation of lipid ROS and ferroptosis, exacerbating pathological outcomes. In SA-AKI, the kidney is considered one of the organs most sensitive to ferroptosis. Due to their high oxygen consumption rate, renal tubular epithelial cells, which are exposed to a hyperoxic environment, are particularly vulnerable to oxidative stress driven by the imbalance between ROS and antioxidants. Ferroptosis, as a lipid peroxidation-dependent cell death mechanism, is propelled by ROS accumulation [9, 10, 24, 25].

Notably, immune cells such as macrophages, T cells, and B cells are also susceptible to ferroptosis, leading to reduced cell number and functionality. Furthermore, ferroptosis cells can be recognized by immune cells, triggering a cascade of inflammatory or specific immune responses. Macrophages, as pivotal innate immune cells and antigen-presenting cells, exhibit remarkable plasticity [26, 27]. On one hand, they initiate innate immune responses by recognizing danger signals in the microenvironment; on the other hand, they modulate host immune responses through differential polarization, forming a multidimensional spectrum of phenotypes to adapt to microenvironmental changes. Thus, macrophages play a critical role in regulating host immune homeostasis and inflammatory responses during sepsis. Moreover, ferroptosis immune cells provide metabolic resources such as iron and fatty acids for bacterial growth, exacerbating the vicious cycle of infection, and ultimately leading to multiple organ dysfunction. High levels of serum iron in septic patients have been positively correlated with mortality, further supporting this phenomenon [28–30].

RP105, also known as Radioprotective 105 kDa protein or CD180, is a type I transmembrane protein initially identified on the surface of murine B cells [31, 32]. It promotes the proliferation and antibody secretion of spleen-derived mature B cells and protects them from apoptosis induced by radiation or dexamethasone. Subsequent studies revealed that RP105 belongs to the Toll-like receptor (TLR) family of pattern recognition receptors [33]. Structurally, the extracellular domain of RP105, comprising 22 leucine-rich repeats (LRRs), shows high homology to TLR4. Functionally, RP105 is closely related to TLR4 and serves as a regulatory molecule for the TLR4 signaling pathway [34, 35]. In vivo, RP105 forms a complex with myeloid differentiation protein 1 (MD-1), which is restrictively expressed on the surface of B cells, dendritic cells, and macrophages [31]. RP105 has been implicated in various diseases, including systemic lupus erythematosus, systemic sclerosis, SA-AKI, myocardial infarction, and atherosclerosis [35–39]. In B cells, RP105 activates proliferation and antibody secretion. However, in macrophages, RP105 downregulates TLR4-mediated inflammatory signaling pathways, reducing cytokine release and exerting a negative regulatory effect. Studies have shown that RP105 overexpression improves ischemia-reperfusion and sepsis-induced acute kidney injury in mice by modulating the TLR4/NF-κB pathway. Furthermore, RP105 suppresses oxidative stress induced by myocardial ischemia-reperfusion injury (MI/RI) through activation of the Lyn/Syk/STAT3 pathway [36, 40]. In addition, we found a significant downregulation of RP105 in sepsis-related datasets, both in human peripheral blood mononuclear cells (PBMCs) stimulated with lipopolysaccharide (LPS) and in peripheral blood samples from sepsis patients compared to healthy controls. Given that RP105 has been shown to modulate immune responses, suppress inflammation, and mitigate oxidative stress in previous studies, we hypothesize that reduced RP105 expression may disrupt immune homeostasis, leading to excessive accumulation of reactive oxygen species (ROS) and aggravated lipid peroxidation, thereby triggering ferroptosis and contributing to renal tubular epithelial cell injury.

In the progression of SA-AKI, ferroptosis and oxidative stress are two critical pathogenic factors. Oxidative stress leads to ROS accumulation, which further promotes lipid peroxidation and ultimately triggers ferroptosis, exacerbating kidney injury. Although the regulatory role of RP105 in this pathological process has not been fully elucidated, we hypothesize that RP105 may inhibit oxidative stress, thereby reducing ROS production, mitigating lipid peroxidation, and slowing the progression of ferroptosis. This study aims to provide a new perspective on how RP105 regulates ferroptosis in sepsis-induced kidney injury.

## Results

### RP105 is downregulated in SA-AKI kidneys with altered ferroptosis levels in sepsis

**Fig. 1:**
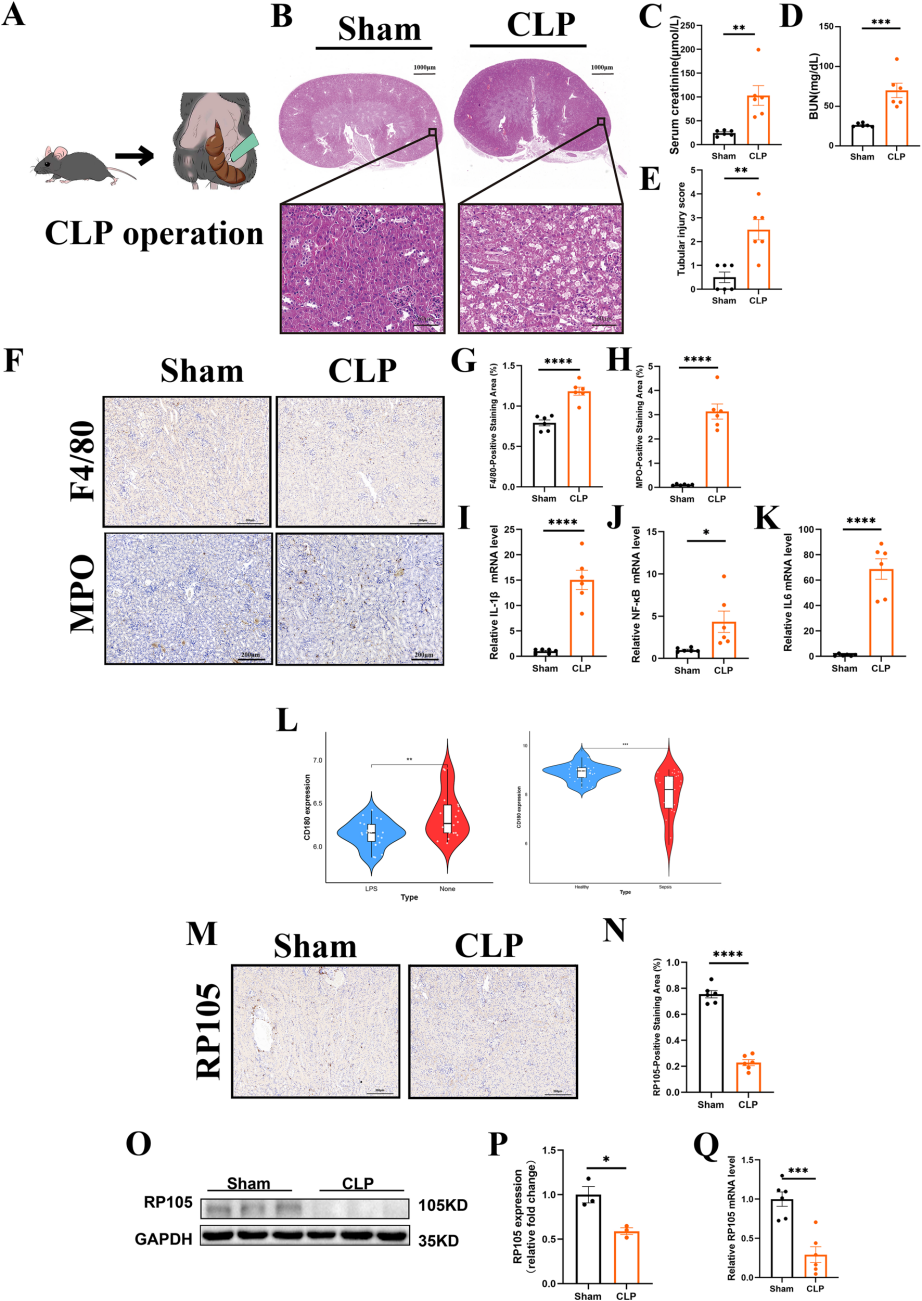
SA-AKI model exhibits renal injury, inflammation, and immune cell infiltration accompanied by downregulation of RP105 expression. **A** Schematic illustration of the CLP-induced SA-AKI mouse model. **B** Representative H&E-stained images of kidney tissue, with the upper panels showing low-magnification views and the lower panels showing magnified areas. **C**, **D** Measurement results of SCr and BUN levels. **E** Tubular injury scoring. **F** Representative immunohistochemical staining images for F4/80 and MPO. **G**, **H** Quantitative analysis of F4/80- and MPO-positive staining areas. **I**−**K** qPCR analysis of mRNA levels for inflammatory genes, including IL-1β, NF-κB, and IL-6. **L** Analysis results from the GSE46955 database using LPS-stimulated human monocytes. **M** Representative immunohistochemical staining images for RP105. **N** Quantitative analysis of RP105-positive staining areas. **O** Western blot analysis of RP105 protein levels. **P** Quantitative analysis of RP105 protein expression levels. (Q) qPCR analysis of RP105 gene mRNA levels. All data are mean ± SEM. *P < 0.05, **P < 0.01, ***P < 0.001, ****P < 0.0001.

We established a mouse model of SA-AKI induced by CLP. Briefly, male mice underwent CLP surgery and were sacrificed 24 h later (Fig. 1A). Kidneys were harvested and processed for formalin fixation and paraffin embedding (FFPE). H&E staining revealed more tubular cast formation and epithelial vacuolar degeneration in the kidneys of the CLP group compared to controls, with significantly higher tubular injury scores (Fig. 1B–E). SCr and BUN levels were significantly elevated in the CLP group, confirming the successful induction of SA-AKI (Fig. 1C, D). Immunohistochemical staining for F4/80 (macrophage marker) and MPO (neutrophil marker) showed increased immune cell infiltration in the CLP group (Fig. 1F–H). Furthermore, the CLP group exhibited elevated levels of inflammation (Fig. 1I–K). These data indicate that kidney injury in the CLP group was exacerbated, accompanied by significant immune cell infiltration and heightened inflammation. To investigate RP105 expression, we analyzed public sepsis-related databases (Fig. 1L, GSE46955 and GSE69063). Bioinformatics analysis of gene expression profiles revealed that RP105 expression was significantly reduced in LPS-stimulated human monocytes compared to the control group in GSE46955 and in peripheral blood samples from sepsis patients in GSE69063. Consistent with these findings, RP105 expression was significantly reduced in the kidney of CLP-treated mice, as confirmed by WB, qPCR, and IHC analyses (Fig. 1M–Q). Additionally, we further demonstrated increased macrophage infiltration in the CLP group through immunofluorescence staining, accompanied by a further reduction in RP105 expression. These findings suggest that RP105 may play a critical regulatory role in sepsis-induced kidney injury, potentially through modulation of immune cells infiltration (Supplementary file, Fig. S1).

### RP105 knockout aggravates kidney injury in SA-AKI

**Fig. 2:**
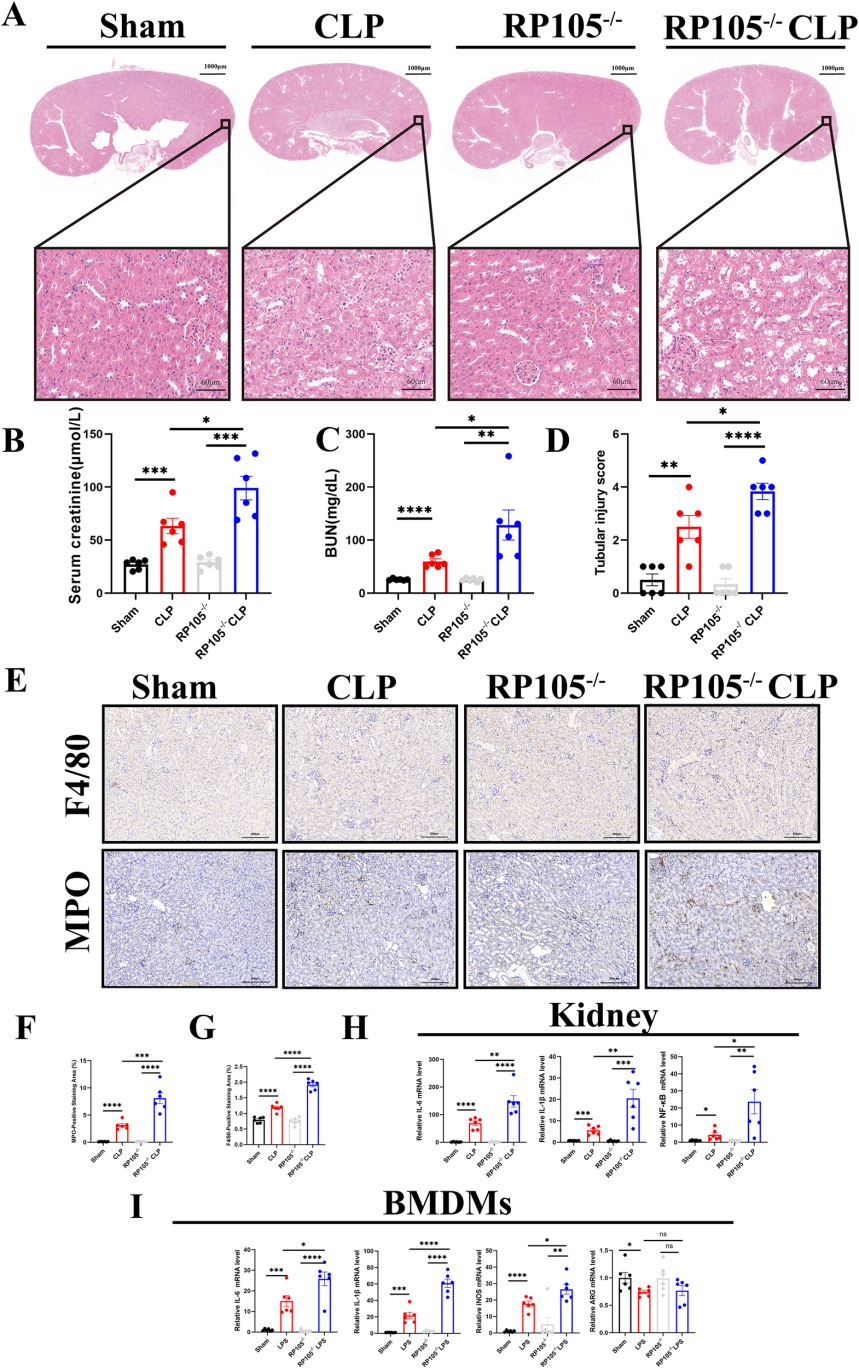
RP105 knockout exacerbates kidney injury and inflammation in SA-AKI. **A** Representative H&E-stained kidney sections from Sham, CLP, RP105^−/−^, and RP105^−/−^ CLP mice, with the upper panels showing low-magnification images and the lower panels showing magnified views. **B**, **C** SCr and BUN measurements. **D** Tubular injury scoring (**E**) Representative immunohistochemical staining images for F4/80 and MPO in kidney sections from each group. **F**, **G** Quantitative analysis of F4/80- and MPO-positive staining areas. **H** qPCR analysis of pro-inflammatory cytokine (e.g., IL-6, IL-1β) and NF-κB mRNA levels in kidney tissue. **I** qPCR analysis of IL-6, IL-1β, iNOS, and ARG1 mRNA levels in bone marrow-derived macrophages (BMDMs). All data are mean ± SEM. *P < 0.05, **P < 0.01, ***P < 0.001, ****P < 0.0001.

To investigate the role of RP105 in SA-AKI, we generated an RP105 global knockout mouse model (Supplementary file, Fig. S2). Knockout efficiency was confirmed by significantly reduced RP105 protein and mRNA levels in kidney tissues, and complete absence of staining on immunohistochemistry (Supplementary file, Fig. S3). Under baseline conditions, no significant differences were observed between Sham and RP105^−/−^ mice in kidney function, histopathology, or immune cell infiltration. However, following CLP treatment, H&E staining revealed a more severe tubular injury in RP105^−/−^ CLP mice, with increased tubular cast formation and exacerbated epithelial vacuolar degeneration (Fig. 2A–D). Additionally, RP105^−/−^ CLP mice exhibited significantly higher serum creatinine and BUN levels compared to CLP mice. Immunohistochemical analysis further demonstrated significantly greater immune cell infiltration in the RP105^−/−^ CLP group compared to the CLP group (Fig. 2E–G). At the molecular level, RP105^−/−^ CLP mice showed significantly elevated mRNA levels of pro-inflammatory cytokines (such as IL-6 and IL-1β), along with increased expression of NF-κB inflammatory signaling molecules, indicating that RP105 deficiency exacerbates the inflammatory response. Additionally, we assessed the expression of relevant inflammatory factors in BMDMs. The results revealed that IL-6, IL-1β, and iNOS levels were increased in the RP105^−/−^ CLP group, whereas ARG1 levels showed no significant difference, suggesting that RP105 primarily affects the pro-inflammatory phenotype (Fig. 2H-I).

### RP105 knockout promotes macrophage infiltration and M1 polarization

**Fig. 3:**
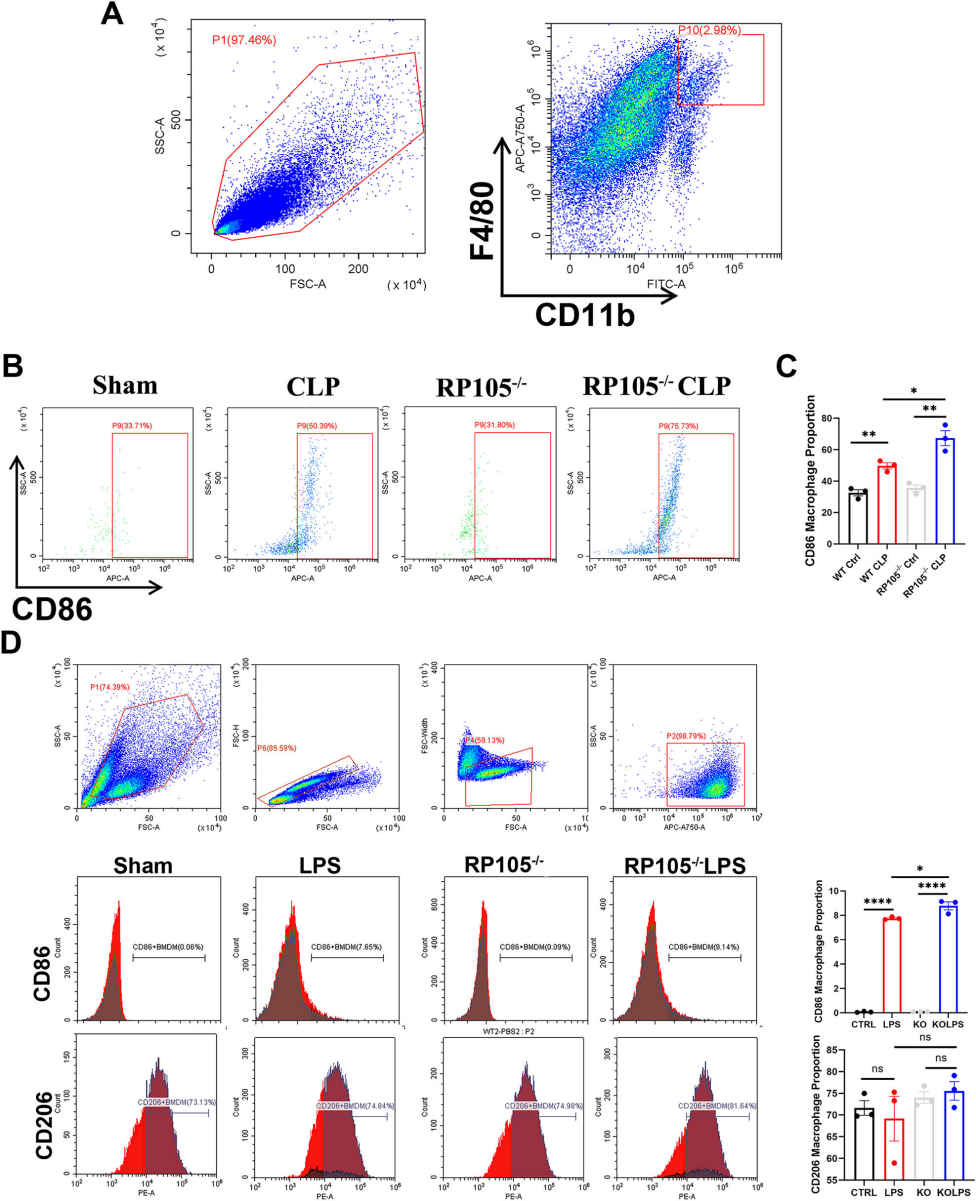
RP105 deficiency enhances macrophage infiltration and promotes M1 polarization in SA-AKI. **A** Representative flow cytometry gating strategy for kidney-infiltrating macrophages. **B** Representative flow cytometry plots of CD86-positive macrophages in Sham, CLP, RP105^−/−^, and RP105^−/−^ CLP groups. **C** Quantitative analysis of the proportion of CD86-positive macrophages. **D** Flow cytometry gating strategy and representative histograms for CD86 and CD206 expression in BMDMs from Sham and RP105^−/−^ mice with or without LPS stimulation, as well as quantitative analysis of the proportion of CD86- and CD206-positive macrophages. All data are mean ± SEM. *P < 0.05, **P < 0.01, ***P < 0.001, ****P < 0.0001.

In our study, flow cytometry revealed a significant increase in macrophage infiltration in the kidneys after CLP treatment, particularly in the RP105^−/−^ CLP group (Fig. 3A–C). RP105 deficiency appeared to promote macrophage recruitment and M1 polarization, highlighting its key role in regulating macrophage polarization and inflammation. After LPS stimulation, RP105^−/−^ BMDMs exhibited a significantly higher proportion of M1 polarization compared to Sham BMDMs, while no significant difference was observed in M2 polarization. These findings are consistent with previous observations and indicate the role of RP105 in inhibiting pro-inflammatory macrophage polarization (Fig. 3D).

### Transcriptomic analysis reveals RP105-regulated ferroptosis pathways in SA-AKI

**Fig. 4:**
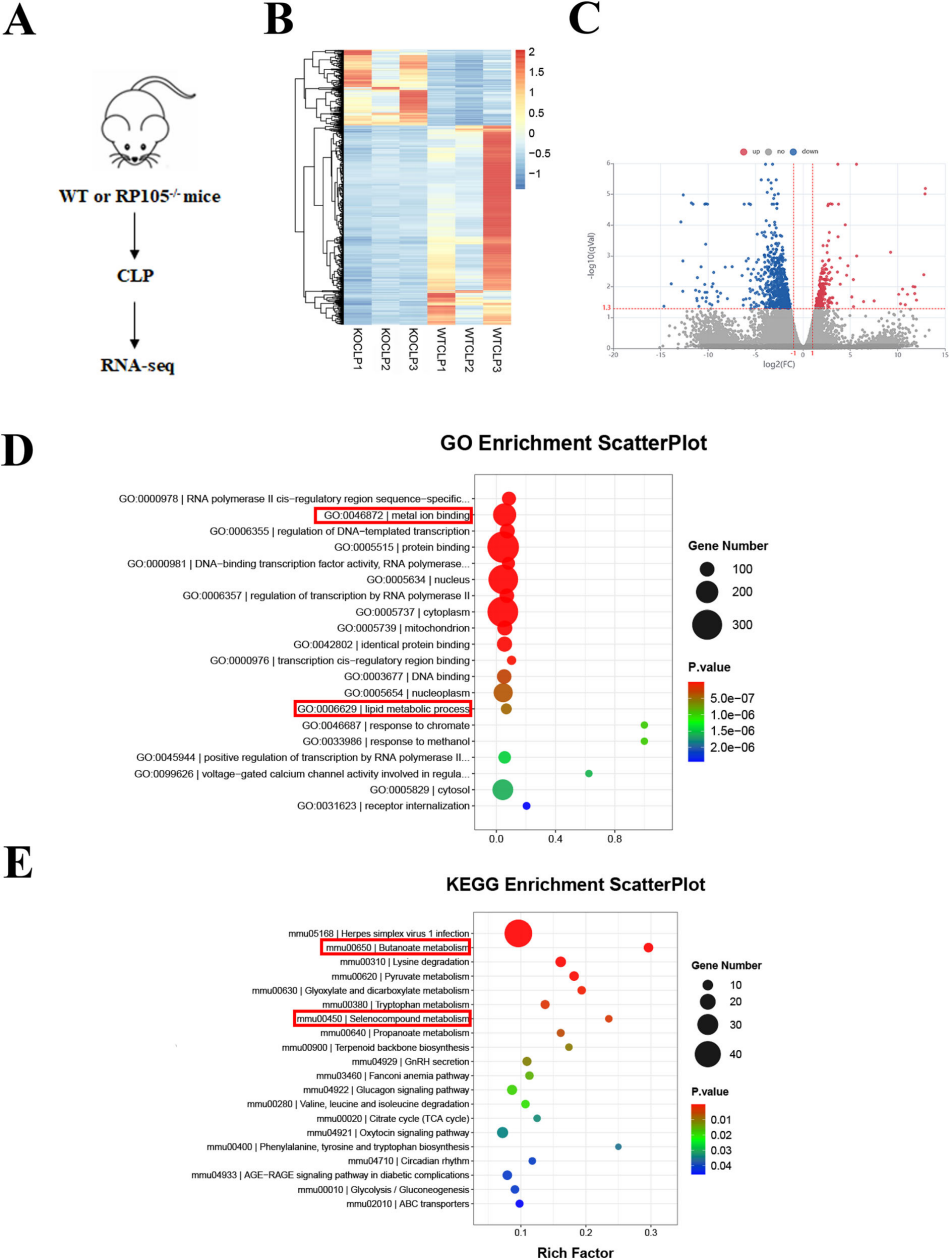
Transcriptomic profiling reveals oxidative stress- and ferroptosis-related pathway enrichment in RP105^−/−^ septic kidneys. **A** Schematic representation of RNA-seq analysis performed on WT and RP105^−/−^ mice after CLP treatment. **B** Heatmap displaying differentially expressed genes between CLP and RP105^−/−^ CLP groups. **C** Volcano plot illustrating upregulated and downregulated genes. **D** GO enrichment scatter plot of differentially expressed genes. **E** KEGG enrichment scatter plot of differentially expressed genes.

**Fig. 5:**
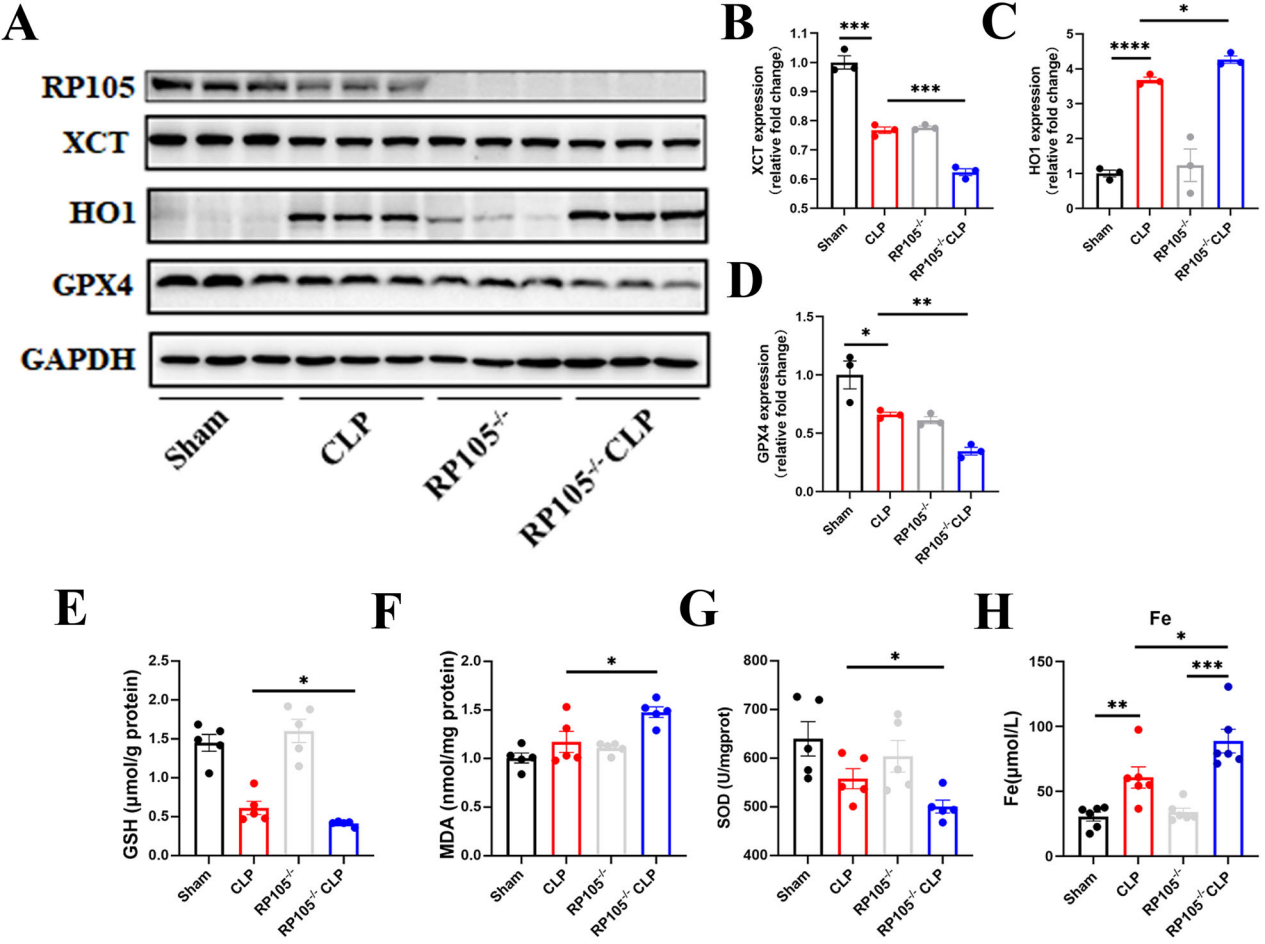
RP105 alleviates ferroptosis in septic kidneys by regulating oxidative stress and iron metabolism. **A** Representative Western blot images for RP105, xCT, HO-1, and GPX4 protein levels in kidney tissues from Sham, CLP, RP105^−/−^, and RP105^−/−^ CLP groups, with GAPDH as a loading control. **B**−**D** Quantitative analysis of xCT, HO-1, and GPX4 protein expression based on Western blot results. **E**−**H** Measurements of GSH, MDA, SOD, and Fe levels in kidney or serum samples. All data are mean ± SEM. *P < 0.05, **P < 0.01, ***P < 0.001, ****P < 0.0001.

To study the molecular mechanisms, we performed transcriptomic sequencing of kidneys from the CLP and RP105^−/−^ CLP mice. The volcano plot and heatmap revealed differentially expressed genes between the two groups (Fig. 4A–C). Further Gene Ontology (GO) enrichment analysis indicated that these differentially expressed genes were mainly enriched in pathways related to oxidative stress, metal ion metabolism, and butyrate metabolism, all of which are closely associated with ferroptosis (Fig. 4D–E). Western blot analysis of renal tissues showed that the expression of ferroptosis-related proteins GPX4 and xCT was significantly reduced in the RP105^−/−^ CLP group, while the expression of HO-1 was significantly increased **(**Fig. 5A–D, and Supplementary file, Fig. S4A**)**. RP105^−/−^ CLP mice also exhibited significantly lower levels of SOD and GSH, along with increased MDA levels. Immunofluorescence staining further confirmed that ROS expression was significantly elevated in the kidneys of RP105^−/−^ CLP mice. These results demonstrate that RP105 alleviates ferroptosis by regulating oxidative stress and iron metabolism. Additionally, the serum iron levels in RP105^−/−^ mice were significantly higher, indicating a disruption in iron metabolism (Fig. 5E–H). Overall, these findings suggest that RP105 plays a crucial role in protecting against sepsis-induced renal injury by inhibiting oxidative stress and ferroptosis, and its deficiency exacerbates these pathological processes.

### RP105 protects mitochondria and inhibits ferroptosis

**Fig. 6:**
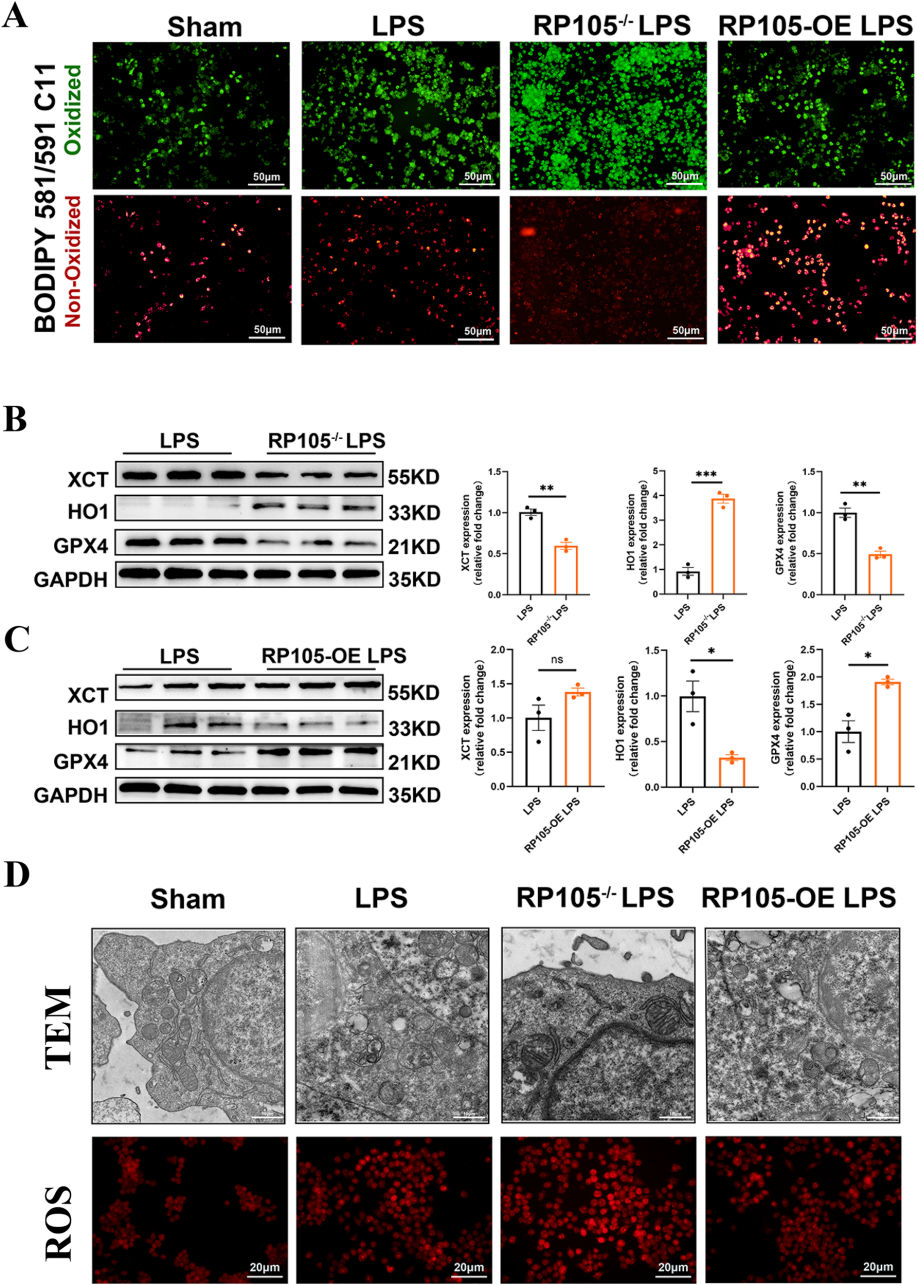
RP105 preserves mitochondrial integrity and inhibits ferroptosis through regulation of oxidative stress and key ferroptotic markers. **A** Representative JC-1 staining images showing changes in mitochondrial membrane potential (red: polarized mitochondria; green: depolarized mitochondria) in Sham, LPS, RP105^−/−^ LPS, and RP105-OE LPS-treated cells. **B**, **C** Western blot analysis of ferroptosis-related proteins (xCT, HO-1, and GPX4) in LPS-treated macrophages from RP105^−/−^ and RP105-OE groups, with quantitative comparisons. **D** Representative transmission electron microscopy (TEM) images showing mitochondrial ultrastructure and corresponding ROS fluorescence images (red fluorescence) in Sham, LPS, RP105^−/−^ LPS, and RP105-OE LPS groups. All data are mean ± SEM. *P < 0.05, **P < 0.01, ***P < 0.001, ****P < 0.0001.

To evaluate the role of RP105 in mitochondrial protection and ferroptosis inhibition, we first assessed mitochondrial membrane potential changes using JC-1 staining (Fig. 6A). Both the control group and RP105-overexpressing cells exhibited stronger red fluorescence (intact mitochondria) and weaker green fluorescence (depolarized mitochondria), indicating that mitochondrial function was maintained. In contrast, after LPS treatment, red fluorescence was significantly reduced, and green fluorescence increased, indicating mitochondrial depolarization and dysfunction. These results suggest that RP105 plays a protective role in maintaining mitochondrial membrane potential and function. Furthermore, RP105 overexpression partially alleviated LPS-induced depolarization, while RP105 knockout exacerbated mitochondrial damage and depolarization.

To further investigate the impact of RP105 on ferroptosis, we analyzed the expression of ferroptosis-related markers HO-1, GPX4, and xCT in macrophages by Western blotting. The results showed that LPS stimulation significantly increased HO-1 expression while downregulating GPX4 and xCT. Further analysis revealed that in the RP105^−/−^ group, LPS treatment resulted in higher HO-1 expression and lower GPX4 and xCT expression compared to the control group. In contrast, in the RP105-overexpressing group, HO-1 expression was lower, while GPX4 and xCT expression were elevated. These findings suggest that RP105 regulates ferroptosis by modulating the expression of HO-1, GPX4, and xCT, thereby inhibiting ferroptosis and oxidative stress (Fig. 6B, C, and Supplementary File, Fig. S4B). Immunofluorescence staining confirmed reduction of GPX4 expression in the RP105^−/−^ group (Supplementary file, Fig. S5). To further study the RP105’s role in mitochondrial dysfunction, we also examined mitochondrial morphology. Transmission electron microscopy revealed more severe mitochondrial damage in RP105^−/−^ cells compared to the control group, including mitochondrial swelling, membrane disintegration, and reduced cristae, all of which are hallmarks of ferroptosis. Additionally, increased ROS fluorescence further supported the role of RP105 in protecting cells from ferroptosis (Fig. 6D).

### Ferrostatin-1 reverses RP105 deficiency-induced ferroptosis and kidney injury

**Fig. 7:**
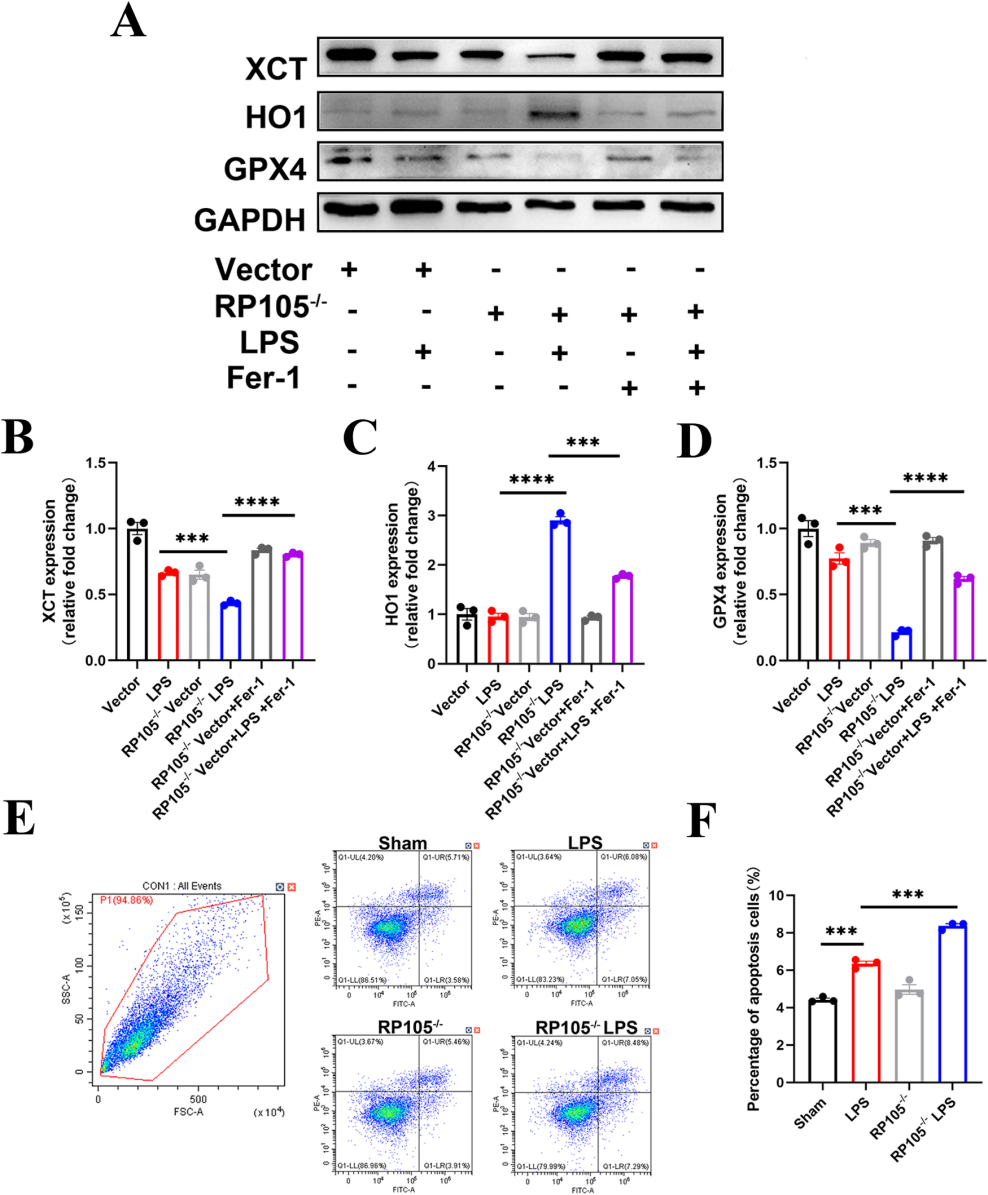
Fer-1 reverses ferroptosis in RP105-deficient macrophages and reduces LPS-induced epithelial cell injury in a co-culture system. **A** Representative Western blot images of xCT, HO-1, and GPX4 expression under different treatments (Vector, RP105^−/−^, LPS, Fer-1). GAPDH was used as a loading control. **B**-**D** Quantitative analysis of xCT, HO-1, and GPX4 protein expression from Western blot data. **E** Representative flow cytometry plots of apoptosis in renal tubular epithelial cells co-cultured with macrophages under indicated treatments. **F** Quantification of apoptotic cell percentage based on flow cytometry analysis. All data are mean ± SEM. *P < 0.05, **P < 0.01, ***P < 0.001, ****P < 0.0001.

Notably, treatment with the ferroptosis inhibitor Ferristatin-1 effectively reversed the ferroptosis process caused by RP105 deficiency, providing further evidence that RP105 protects cells from damage through the inhibition of ferroptosis (Fig. 7A–D). In a co-culture model of renal tubular epithelial cells, HK-2, and macrophages, we found that after 24 h of LPS treatment, RP105^−/−^ significantly exacerbated apoptosis in renal tubular epithelial cells. This suggests that RP105 plays a key role in regulating ferroptosis in macrophages, and its deficiency worsens LPS-induced ferroptosis, further exacerbating cell damage (Fig. 7E, F).

**Fig. 8:**
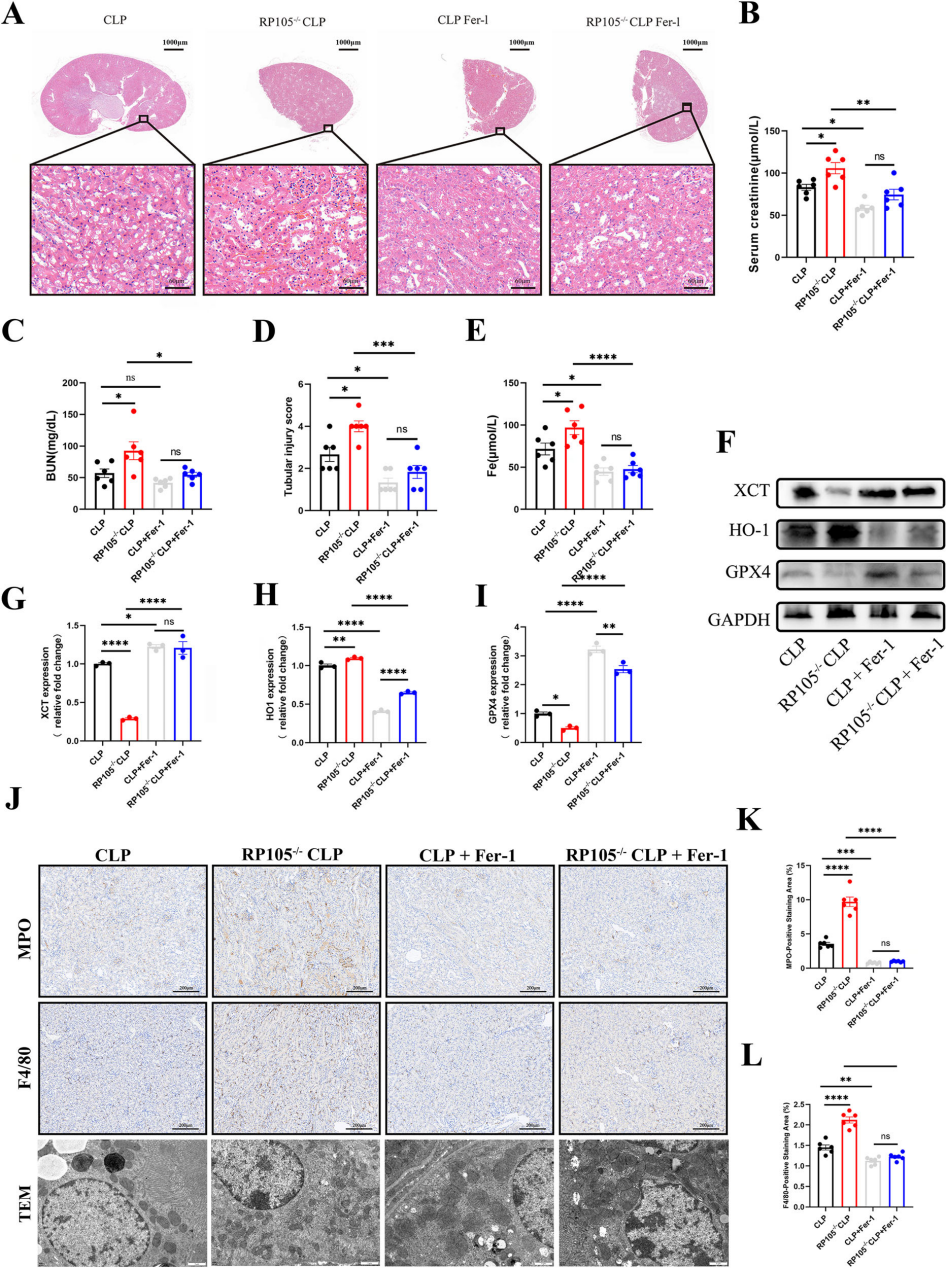
RP105 deficiency aggravates ferroptosis in septic kidneys, which is mitigated by the ferroptosis inhibitor Fer-1. **A** Representative H&E-stained kidney sections from Sham, CLP, RP105^−/−^ CLP, CLP + Fer-1, RP105^−/−^ CLP + Fer-1 mice, with the upper panels showing low-magnification images and the lower panels showing magnified views. **B**, **C** SCr and BUN measurements. **D** Tubular injury scoring. **E** Measurements of Fe levels in kidney or serum samples. **F**−**I** Representative Western blot images of xCT, HO-1, and GPX4 expression under different treatments. **J** Representative immunohistochemical staining images for F4/80 and MPO in kidney sections from each group, and representative transmission electron microscopy (TEM) images showing mitochondrial ultrastructure. **K**, **L** Quantitative analysis of F4/80- and MPO-positive staining areas.

To further validate this hypothesis in vivo, we conducted animal experiments using the CLP model. Mice were divided into four groups: CLP, RP105^−/−^ CLP, CLP+Fer-1, and RP105^−/−^ CLP+Fer-1. Consistent with the above findings, the RP105^−/−^ + CLP group exhibited the most severe renal injury, with elevated BUN, SCr, and serum iron levels, and worsened histopathological and immunohistochemical changes, compared with the CLP group (Fig. 8A–E). Furthermore, administration of the ferroptosis inhibitor Fer-1 markedly alleviated renal injury, with the CLP+Fer-1 group showing the mildest damage, followed by the RP105^−/−^ + CLP + Fer-1 group.

To further verify whether RP105 mitigates sepsis-induced kidney injury by regulating ferroptosis, renal tissues were collected from the above animal models, and the protein expression levels of ferroptosis-related markers HO-1, GPX4, and xCT were evaluated via Western blotting. The results showed that HO-1 expression was significantly upregulated, while GPX4 and xCT levels were markedly downregulated in the RP105^−/−^ + CLP group compared with the CLP group, suggesting that RP105 deficiency may exacerbate ferroptosis. Notably, these changes were partially reversed following Fer-1 administration, with the CLP+Fer-1 group exhibiting the lowest HO-1 expression and the highest GPX4 and xCT levels, consistent with the most attenuated renal injury observed histologically (Fig. 8F–I). TEM was employed to examine ferroptosis-associated mitochondrial alterations in renal tissues (Fig. 8J). In the CLP group, mitochondria exhibited moderate structural damage, including partial cristae loss and rupture of the outer membrane. These changes were more pronounced in the RP105^−/−^ + CLP group, where mitochondria showed severe shrinkage, increased membrane density, and nearly complete cristae loss-hallmark ultrastructural features of ferroptosis. Following treatment with the ferroptosis inhibitor Fer-1, mitochondrial injury was significantly alleviated. The CLP+Fer-1 group displayed the most preserved mitochondrial morphology, while the RP105^−/−^ + CLP + Fer-1 group showed partial structural restoration, further supporting the role of RP105 in attenuating ferroptosis-induced mitochondrial damage in septic kidneys. Immunohistochemical analysis further demonstrated significantly greater immune cell infiltration in the RP105^−/−^ CLP group compared to the CLP group, which was markedly reversed by Fer-1 treatment (Fig. 8J–L).

## Discussion

In this study, we identified a significant downregulation of RP105 expression in human blood-derived monocytes stimulated with LPS through bioinformatics analysis. To further investigate the role of oxidative stress and ferroptosis in sepsis, we employed an RP105^−/−^ mice model. The results demonstrated that sepsis-induced prominent inflammatory responses, intense oxidative stress, ROS release, intracellular iron accumulation, lipid peroxidation, and upregulation of HO-1, accompanied by a marked downregulation of XCT and GPX4 expression. This mechanism exacerbated ferroptosis through immune cell infiltration, particularly by macrophages, which further contributed to kidney injury in sepsis. Additionally, transcriptomic analysis of CLP and RP105^−/−^ CLP mice revealed enrichment in pathways related to oxidative stress, metal ion metabolism, and butyrate metabolism. Butyrate has been shown to inhibit xCT expression and affect xCT-dependent glutathione synthesis. Our findings also indicated that RP105 deficiency significantly increased immune cell infiltration, exacerbating oxidative stress, lipid peroxidation, and ferroptosis. Notably, RP105 KO mice exhibited more severe kidney injury in an SA-AKI model, suggesting a protective role of RP105 in regulating oxidative stress and ferroptosis to mitigate SA-AKI. Moreover, the use of the ferroptosis inhibitor Fer-1 effectively reversed ferroptosis-associated damage caused by RP105 deficiency, further supporting the protective function of RP105. These findings indicate that RP105 may serve as a novel therapeutic target for preventing and treating sepsis-associated AKI by modulating immune cell infiltration, oxidative stress, and ferroptosis, particularly through the regulation of the HO-1/SLC7A11/GPX4 axis.

Infection and inflammation severely disrupt the body’s antioxidant systems, leading to dysregulation of iron metabolism, lipid metabolism, and mitochondrial function, ultimately inducing ferroptosis. In sepsis, a typical systemic infection, elevated inflammatory cytokines [41], aggravated oxidative stress [42], disrupted iron homeostasis [43], and perturbed lipid metabolism collectively contribute to ferroptosis [9, 10, 24, 44]. Ferroptosis is closely associated with sepsis. Previous studies have shown that systemic iron homeostasis alterations in sepsis patients correlate positively with SOFA scores and mortality [45]. Our study further confirms that sepsis-induced AKI is associated with ferroptosis, which exacerbates inflammation and oxidative stress in renal tissues. RP105 plays a critical immunoregulatory role by modulating inflammation and oxidative stress. It has been reported to suppress the expression of NF-κB, iNOS, IL-1β, IL-6, and TNF-α [36]. RP105 achieves this by regulating signaling pathways such as TLR2/4 and PI3K, thereby attenuating excessive inflammatory responses and oxidative stress and reducing tissue damage [33, 46]. This regulatory mechanism is pivotal in controlling the polarization of macrophages between pro-inflammatory M1 and anti-inflammatory M2 phenotypes. Currently, there are no studies investigating RP105’s role in macrophage polarization and ferroptosis. Our research demonstrates that RP105 deficiency significantly promotes the transition of macrophages to the pro-inflammatory M1 phenotype.

In the context of sepsis-induced AKI, RP105 deficiency orchestrates a complex interaction network involving ferroptosis, oxidative stress, and macrophage polarization. These processes form a vicious cycle, which is a positive feedback loop to accelerate tissue damage. RP105 deficiency compromises the regulatory capacity of macrophages to mitigate inflammation and oxidative stress, leading to iron dysregulation and lipid peroxidation, thereby inducing ferroptosis. Metabolic by-products of ferroptosis, such as free iron and ROS, exacerbate oxidative stress, which, in turn, drives macrophage polarization towards the M1 phenotype. Excessive inflammatory responses result in severe tissue damage, oxidative stress, and intracellular iron overload. This expansion of the labile iron pool catalyzes lipid peroxidation via the Fenton reaction, causing organelle damage and cell death, thereby releasing excessive intracellular iron, further promoting ferroptosis and oxidative stress.

Pro-inflammatory cytokines (e.g., IL-6) secreted by M1 macrophages aggravate tubular epithelial cell damage and amplify ferroptosis, forming a positive feedback loop of inflammation and ferroptosis. Additionally, macrophages contribute to renal tissue damage by secreting inflammatory factors and remodeling the immune microenvironment, further exacerbating local inflammation and affecting the function of other cell types. However, the causal relationships and regulatory mechanisms underlying these processes require further investigation.

Ferroptosis-specific inducers, such as Erastin, inhibit the xCT transporter of the Xc⁻ system, deplete intracellular cysteine, and reduce GSH levels [47]. As the most abundant endogenous antioxidant, GSH is a critical free radical scavenger and a key regulator of GPX4 [48]. Depletion of intracellular GSH leads to uncontrolled lipid peroxidation and ferroptosis [49]. GPX4, a glutathione-dependent lipid hydroperoxidase, prevents ferroptosis by converting lipid hydroperoxides into non-toxic lipid alcohols [9, 50]. HO-1 regulates iron homeostasis, antioxidative responses, and anti-inflammatory effects by metabolizing heme to release iron, biliverdin, and carbon monoxide. It plays a dual role in cellular protection and ferroptosis. A study on single-cell datasets from sepsis highlighted significantly elevated HO-1 expression within the monocyte lineage [51]. However, excessive activation of HO-1 can release an abundance of free iron (Fe²⁺), which generates ROS via the Fenton reaction, exacerbating lipid peroxidation and ferroptosis. Particularly under high-inflammatory conditions such as sepsis, aberrant upregulation of HO-1 disrupts iron homeostasis and exacerbates cellular damage [24, 33].

The TLR4/NF-κB signaling pathway plays an important role in ferroptosis-related pathology by promoting both inflammation and ferroptosis. NF-κB not only induces the expression of various pro-inflammatory cytokines but directly binds to the HMOX1 promoter, thereby upregulating HO-1 expression [52]. Accordingly, RP105 is proposed to limit HMOX1 expression by negatively regulating the TLR4/NF-κB pathway, thereby reducing HO-1-mediated iron accumulation and ROS generation, and ultimately attenuating ferroptosis under inflammatory conditions. In addition to NF-κB-mediated activation, HO-1 expression is intricately regulated by multiple transcription factors. Among them, the Nrf2-Bach1 axis represents one of the core regulatory mechanisms. Under physiological conditions, Bach1 binds to the antioxidant response element within the HMOX1 promoter, thereby repressing its transcription. Upon oxidative stress or heme accumulation, Bach1 dissociates, and Nrf2 is released from the Keap1 complex, allowing it to translocate into the nucleus and bind to the ARE, where it activates HO-1 transcription [52].

In addition, existing studies have shown that RP105 can attenuate excessive inflammatory responses and oxidative stress, thereby reducing tissue damage, in part by modulating the PI3K signaling pathway [46]. As Nrf2 is a ferroptosis-sensitive transcription factor, RP105 may also indirectly enhance Nrf2 activity and promote its nuclear translocation by alleviating the TLR4-mediated suppression of the PI3K/Akt signaling axis [18, 53, 54]. Through this mechanism, RP105 may regulate an additional antioxidant program closely linked to ferroptosis, further contributing to cellular protection under oxidative stress.

RP105 may exert a protective effect in sepsis-associated acute kidney injury (SA-AKI) by mitigating oxidative stress and ferroptosis through the mechanisms described above. Specifically, RP105 helps protect renal cells from sepsis-induced ferroptosis by maintaining the expression of GPX4 and xCT, while suppressing the abnormal upregulation of HO-1, thereby reducing intracellular iron accumulation, lipid peroxidation, and oxidative stress.

In our study, RP105 appeared to protect renal cells from sepsis-induced oxidative stress and ferroptosis by maintaining GPX4 and xCT expression levels and suppressing aberrant HO-1 activation, thereby reducing intracellular iron accumulation, lipid peroxidation, and oxidative stress. The regulatory role of RP105 not only highlights its significance in oxidative stress control but also identifies it as a potential molecular target for the prevention and treatment of sepsis-associated AKI.

### Limitations

This study has several limitations. First, RP105^−/−^ mice used in this study were whole-body knockout models, making it difficult to distinguish the cell-type-specific effects of RP105. Future studies should utilize macrophage-specific RP105^−/−^ models to clarify its protective role in macrophage-mediated CLP-induced ferroptosis and kidney injury. Second, this study did not directly investigate the impact of RP105 on renal tubular epithelial cells via macrophage regulation. Co-culture models of macrophages and renal tubular epithelial cells could more accurately simulate immune infiltration and further elucidate the specific mechanisms underlying macrophage-induced renal tissue damage and repair. Additionally, as a transmembrane protein, RP105’s function is regulated not only at the gene expression level but also through post-translational modifications (e.g., N-glycosylation, ubiquitination/deubiquitination) and epigenetic mechanisms (e.g., DNA methylation, histone acetylation). These upstream regulatory mechanisms were not explored in this study, limiting a comprehensive understanding of its role in renal injury and repair. Future studies should consider using macrophage-epithelial cell co-culture systems to better simulate macrophage-mediated immune infiltration in renal tissues. Additionally, investigating the interactions of RP105 among different cell types and its dynamic changes in the renal injury microenvironment will provide a more holistic understanding of its protective role in renal injury.

## Conclusion

This study demonstrates that RP105 plays a pivotal protective role in SA-AKI. RP105 deficiency exacerbates macrophage-induced inflammation, oxidative stress, and ferroptosis, forming a vicious cycle that leads to more severe renal injury. RP105 mitigates tissue damage by regulating the HO-1/SLC7A11/GPX4 axis, inhibiting ferroptosis, and preventing macrophage polarization to the pro-inflammatory phenotype. Despite revealing the critical functions of RP105, further research is needed to elucidate its cell-specific roles and upstream regulatory mechanisms. RP105 holds promise as a novel therapeutic target for sepsis-associated kidney injury.

## Methods

### Animal and sepsis-associated acute kidney injury model

WT mice were purchased from GemPharmatech, and RP105^−/−^ mice were generated through CRISPR/Cas9 gene editing [55]. Mice were housed in a pathogen-free environment under standard conditions (24 ± 2 °C, 50 ± 5% humidity). All animal experiments were performed following the guidelines set by the Animal Care and Ethics Committee of Wuhan University. A CLP procedure was performed to induce a SA-AKI model in 6−8 week-old male mice weighing 20-25 g [56]. Mice were randomly divided into six groups (Sham, CLP, RP105^−/−^, RP105^−/−^CLP, CLP + Ferrostatin-1 (Fer-1), and RP105^−/−^CLP + Fer-1). The group size was determined based on commonly used standards in similar animal studies and prior literature on sepsis-associated acute kidney injury (*n* = 6) [57]. Each mouse was numbered to achieve a single-blind. The sham-operated mice underwent the same surgical procedure without cecal ligation and puncture. Mice were sacrificed 24 h post-CLP or sham operation to collect serum and kidney tissues for subsequent analyses. Fer-1 (5 mg/kg, MCE, HY-100579) was dissolved in DMSO and diluted in sterile saline. It was administered intraperitoneally (i.p.) at a single dose 1 h before CLP surgery to inhibit ferroptosis in vivo [58]. All in vivo experiments were conducted in accordance with Chinese legislation on the care and use of laboratory animals and were approved by the Animal Care and Use Committee of Wuhan University.

### Cell culture

#### Bone Marrow-Derived Macrophages (BMDMs)

Under sterile conditions, bone marrow cells were harvested by flushing the femurs and tibias of Sham and RP105^−/−^ C57BL/6 J mice with sterile phosphate-buffered saline (PBS). The cells were resuspended in DMEM medium (Gibco) supplemented with 10% fetal bovine serum (FBS, Gibco), 100 IU/mL penicillin, and 100 μg/mL streptomycin. The cell suspension was seeded into culture plates, and 100 ng/mL macrophage colony-stimulating factor (M-CSF, MedChemExpress) was added. Cells were incubated at 37 °C in a humidified atmosphere containing 5% CO₂. The medium was refreshed every 2–3 days with supplementation of M-CSF. After 7 days of induction, bone marrow cells were fully differentiated into mature BMDMs. Adherent BMDMs were collected and treated with 1 μg/mL lipopolysaccharide (LPS, MCE) for subsequent experiments [59, 60].

#### RAW264.7 cells

The RAW264.7 murine macrophage cell line was obtained from the China Center for Type Culture Collection, CCTCC, Wuhan University. Cells were cultured in DMEM medium supplemented with 5% FBS. Transfection was performed using the SuperKine™ Lipo30.0 reagent (Abbkine) according to the manufacturer’s instructions. Plasmids for transfection, including RP105 overexpression constructs were supplied by GeneChem Co., Ltd., and RP105 knockout cell lines were generated by Ubigene. RAW264.7 were collected and treated with 1 μg/mL lipopolysaccharide (LPS, MCE) for subsequent experiments and RAW264.7 were treated with Fer-1 (5 μM; MCE) for 24 h to inhibit ferroptosis following LPS stimulation.

#### Human renal proximal tubule epithelial cells (PTECs, HK-2)

The HK-2 cell line was obtained from the China Center for Type Culture Collection at the College of Life Sciences, CCTCC, Wuhan University. Cells were cultured in DMEM/F12 medium (HyClone) supplemented with 10% FBS and incubated in 10 cm culture dishes at 37 °C in a 5% CO₂ incubator.

#### Co-culture of macrophages and HK-2 cells

Co-culture experiments were performed using Transwell inserts. RAW264.7 macrophages were seeded into the Transwell inserts, which were then placed into culture dishes containing HK-2 cells, forming an upper chamber with macrophages and a lower chamber with HK-2 cells. LPS (1 μg/mL, MCE) was added to the co-culture system to induce an inflammatory response in macrophages, indirectly affecting HK-2 cells. Post-intervention, HK-2 cells were collected and apoptosis was analyzed using flow cytometry to evaluate the impact of macrophage-mediated inflammation on HK-2 cell survival [61].

#### Serum biomarkers of kidney injury and iron content

Serum creatinine (SCr) and blood urea nitrogen (BUN) levels were measured using commercial kits from Nanjing Jiancheng Bioengineering Institute. Absorbance at 546 nm was recorded with a microplate reader (Thermo Fisher) to quantify SCr and BUN concentrations. Serum iron levels were determined using a total iron detection kit (Servicebio, G4301-48T).

#### Renal histology

Kidney tissue sections were stained with hematoxylin and eosin (H&E) for histological analysis. Tubular injury scores were quantified based on the percentage of damaged tubules: 1 = no injury; 2 = < 25%; 3 = 25–49%; 4 = 50–74%; 5 = ≥ 75% [62].

### Immunofluorescence staining

Immunofluorescence staining was performed as described in reference [62]. Sections were incubated with primary antibodies against F4/80 (5–10 µg/mL, Abcam, ab6640), RP105 (1:100, Abcam, ab184956), and GPX4 (1:100, Abcam, ab125066) overnight at 4 °C. Images were captured using a fluorescence microscope, and at least six fields per section were analyzed for the percentage of positive cells.

#### Immunohistochemical staining

Paraffin-embedded kidney sections (4 µm thick) were deparaffinized and subjected to antigen retrieval using ethylenediaminetetraacetic acid (EDTA). Sections were incubated overnight at 4 °C with primary antibodies against F4/80 (1:5000, Abcam, ab300421) and myeloperoxidase (MPO, 1:1000, Abcam, ab208670). A secondary antibody (Goat Anti-Rabbit IgG H&L, 1:500, Abcam, ab97051) was applied for 1 h at room temperature. Images were captured using a 3DHISTECH P250 FLASH III automated digital slide scanner and analyzed with ImageJ.

#### Transmission Electron Microscopy (TEM)

RAW264.7 macrophages were fixed in 2.5% glutaraldehyde overnight, followed by post-fixation in 2% osmium tetroxide. Samples were dehydrated with graded ethanol and embedded in epoxy resin. Ultrathin sections (70–90 nm) were stained with uranyl acetate and lead citrate and observed using a Tecnai G2 20 TWIN transmission electron microscope (FEI, USA) at 200 kV.

### Western blot

Samples were lysed in RIPA buffer, centrifuged, and supernatants were collected. Equal amounts of protein were separated by SDS-PAGE and transferred to PVDF membranes. Membranes were blocked with 5% bovine serum albumin and incubated overnight at 4 °C with primary antibodies against RP105 (Abcam, ab184956), GPX4 (Abmart, TD6701), HO-1 (Abmart, TA5393), and XCT (Abmart, T57046). Secondary antibodies (1:5000 dilution) were applied for 2 h at room temperature, and bands were visualized using a chemiluminescence imaging system (Tanon-5200, Tanon Science and Technology Co., Ltd, China).

#### Relative Gene Expression Analysis (RT-qPCR)

Total RNA was extracted from tissues using Trizol reagent, and cDNA was synthesized using a reverse transcription kit (TOYOBO) following the manufacturer’s protocol. RT-qPCR was performed using SYBR qPCR Master Mix (Cwbio). Gene expression was normalized to GAPDH as an internal control. Primer sequences are listed in Supplementary Table S1.

#### Transcriptomic sequencing

Whole transcriptome analysis was performed on kidney tissues from Sham and RP105^−/−^ mice subjected to sepsis modeling, with three biological replicates per group. RNA was extracted using the Trizol reagent, and RNA-seq libraries were prepared and sequenced on the OmicStudio platform. Differentially expressed genes were identified, and heatmaps and enrichment analyses were generated.

#### ROS levels and mitochondrial membrane potential

ROS activity was assessed by measuring the fluorescence intensity of dihydroethidium (DHE, BestBio, BB-4705), malondialdehyde (MDA, Beyotime, S0131S), and superoxide dismutase (SOD, Nanjing Jiancheng Bioengineering Institute, A001-3-2) levels. Glutathione (GSH) levels were measured using a kit (Nanjing Jiancheng, A006-2-1). Mitochondrial membrane potential was evaluated using a JC-1 assay kit (BBcellProbe®, BB-4105). Fluorescence microscopy was used to record imagess [63].

#### Flow cytometry

Tissue samples were minced and digested in 2 mg/mL type I collagenase in DMEM at 37 °C for 30 min. Single-cell suspensions were filtered through a 40 μm cell strainer. Cells were stained with antibodies for CD11b-FITC (myeloid cells; Invitrogen/eBioscience, Cat# 11-0112-82, Clone: M1/70), F4/80-APC-Cy7 (macrophages; BioLegend, Cat# 123118, Clone: BM8), CD86-APC (activated macrophages and dendritic cells; BioLegend, Cat# 105012, Clone: GL-1), and CD206-PE (M2 macrophages; BioLegend, Cat# 141705, Clone: C068C2). Flow cytometric analysis was performed using a Beckman flow cytometer [64]. Apoptotic cells were determined by flow cytometry assay using Annexin V-FITC/PI Double-Staining Apoptosis Detection Kit (BestBio, Shanghai, People’s Republic of China).

### Transcriptomic data analysis

Two publicly available transcriptome datasets were downloaded from the Gene Expression Omnibus (GEO) database: GSE69063 (peripheral blood samples from sepsis patients and healthy controls) and GSE46955 (LPS-stimulated human peripheral blood mononuclear cells (PBMCs)). The raw data were preprocessed and normalized using R, and differential expression analysis was performed using the limma package. Subsequently, the expression levels of CD180 were extracted from both datasets and intergroup expression differences were assessed using the Wilcoxon rank-sum test.

### Statistical analysis

Data were analyzed using GraphPad Prism 9.0 and presented as mean ± SEM. Statistical significance between groups was determined using Student’s *t*-test, with a *P*-value < 0.05 considered statistically significant. Comparisons among more than two groups were conducted using a one-way analysis of variance (ANOVA) followed by Tukey’s post hoc test. The normality of data distribution was assessed using the Shapiro-Wilk and Kolmogorov-Smirnov tests in GraphPad Prism.

## Supplementary information

Supplementary file

Supplementary file, Fig. S1

Supplementary file, Fig. S2

Supplementary file, Fig. S3

Supplementary file, Fig. S4

Supplementary file, Fig. S5

Original Data

Supplementary Table S1

## Data Availability

The RNA sequence data of this manuscript are deposited and available upon request to the corresponding author at: phq2012@whu.edu.cn.
