# Correction: Modulatory role of radioprotective 105 in mitigating oxidative stress and ferroptosis via the HO-1/SLC7A11/GPX4 axis in sepsis-mediated renal injury

**DOI:** 10.1038/s41420-025-02668-6

**Published:** 2025-08-26

**Authors:** Hong Duo, Yanwei Yang, Jun Luo, Yumeng Cao, Qian Liu, Jiarui Zhang, Siqi Du, Jian You, Guqin Zhang, Qifa Ye, Huaqin Pan

**Affiliations:** 1 National Quality Control Center for Donated Organ Procurement, Hubei Key Laboratory of Medical Technology on Transplantation, Hubei Clinical Research Center for Natural Polymer Biological Liver, Hubei Engineering Center of Natural Polymer-Based Medical Materials, Zhongnan Hospital of Wuhan University, Institute of Hepatobiliary Diseases of Wuhan University, Transplant Center of Wuhan University, Wuhan, Hubei China; 2 Department of Critical Care Medicine, Zhongnan Hospital of Wuhan University, Clinical Research Center of Hubei Critical Care Medicine, Wuhan, 430071 China; 3 Nanchang Hongdu Hospital of Traditional Chinese Medicine, 330006 Jiangxi, China; 4 College of Life Sciences, Wuhan University, Wuhan, 430072 Hubei China; 5 Department of Respiratory and Critical Care Medicine, Zhongnan Hospital of Wuhan University, Wuhan, 430071 China; 6 Zhongnan Hospital of Wuhan University, Institute of Hepatobiliary Diseases of Wuhan University, Transplantation Intensive Care Unit, Transplant Center of Wuhan University, Hubei Key Laboratory of Medical Technology on Transplantation, Wuhan, 430071, China; Department of Critical Care Medicine, Zhongnan Hospital of Wuhan University, Clinical Research Center of Hubei Critical Care Medicine, Wuhan, 430071 China

**Keywords:** Immune cell death, Cell death, Acute kidney injury

Correction to: *Cell Death Discovery* 10.1038/s41420-025-02578-7, published online 01 July 2025

In the published article, the name of one of the authors was incorrectly spelled as “Guqing Zhang”. The correct spelling is “Guqin Zhang.”

The original article has been corrected.

