# Supplementary material for: Modulatory role of radioprotective 105 in mitigating oxidative stress and ferroptosis via the HO-1/SLC7A11/GPX4 axis in sepsis-mediated renal injury
Source: Cell Death Discov. 2025 Jul 1;11:290. doi: 10.1038/s41420-025-02578-7 (PMC12217763; doi:10.1038/s41420-025-02578-7)
Supplement: Supplementary file 1 — Supplementary file [file 41420_2025_2578_MOESM1_ESM.docx]

**Fig. S1. RP105 expression is reduced in the kidneys of CLP-induced SA-AKI mice and is associated with increased macrophage infiltration.**

(A) Representative immunofluorescence images of kidney sections from Sham and CLP groups, stained with DAPI (blue), F4/80 (red), and RP105 (green). The merged images show co-localization and spatial distribution of immune cells and RP105 expression.

**Fig. S2. Genotyping confirmation of RP105 knockout mice.**

(A) Representative PCR genotyping results for wild-type (WT) and RP105 knockout (KO) mice. The size of amplified bands distinguished WT and KO alleles: 418 bp for the WT allele and 334 bp for the KO allele.

**Fig. S3. Immunohistochemical validation of RP105 knockout in mouse kidney tissues.**

(A) Representative immunohistochemical staining images of RP105 in kidney tissues from Sham and RP105^⁻/⁻^ mice.

**Fig. S4. RP105 modulates ferroptosis-related protein expression in renal tissues and macrophages.**

(A) Representative Western blot and quantitative analysis of xCT, HO-1, and GPX4 protein expression in kidney tissues from Sham and CLP groups. (B) Western blot and quantification of ferroptosis-related proteins in RAW264.7 macrophages treated with LPS. All data are mean ± SEM. *P < 0.05, **P < 0.01, ***P < 0.001, ****P < 0.0001.

**Fig. S5. RP105 deficiency reduces GPX4 expression in macrophages under LPS stimulation.**

(A) Representative immunofluorescence staining images of GPX4 (red) and DAPI (blue) in RAW264.7 macrophages from Sham, LPS-treated, RP105^⁻/⁻^, and RP105^⁻/⁻^ + LPS groups. Merged images show co-localization.

**Supplementary Table S1**

Primer sequences used for qRT-PCR analysis.
