# Supplementary figures and images for: Modulatory role of radioprotective 105 in mitigating oxidative stress and ferroptosis via the HO-1/SLC7A11/GPX4 axis in sepsis-mediated renal injury
Source: Cell Death Discov. 2025 Jul 1;11:290. doi: 10.1038/s41420-025-02578-7 (PMC12217763; doi:10.1038/s41420-025-02578-7)

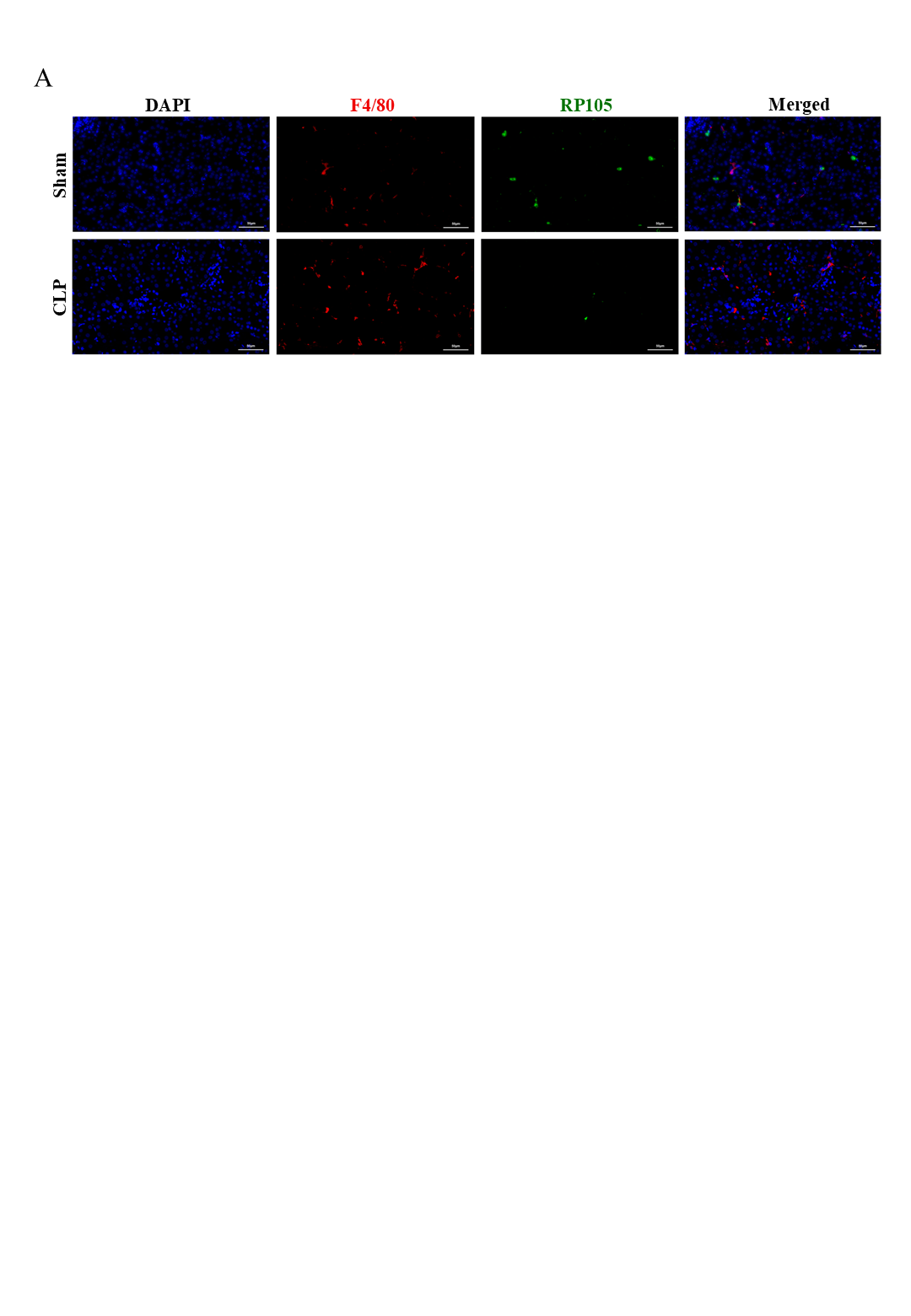

Supplement: Supplementary file 2 — Supplementary file, Fig. S1 [file 41420_2025_2578_MOESM2_ESM.tif]

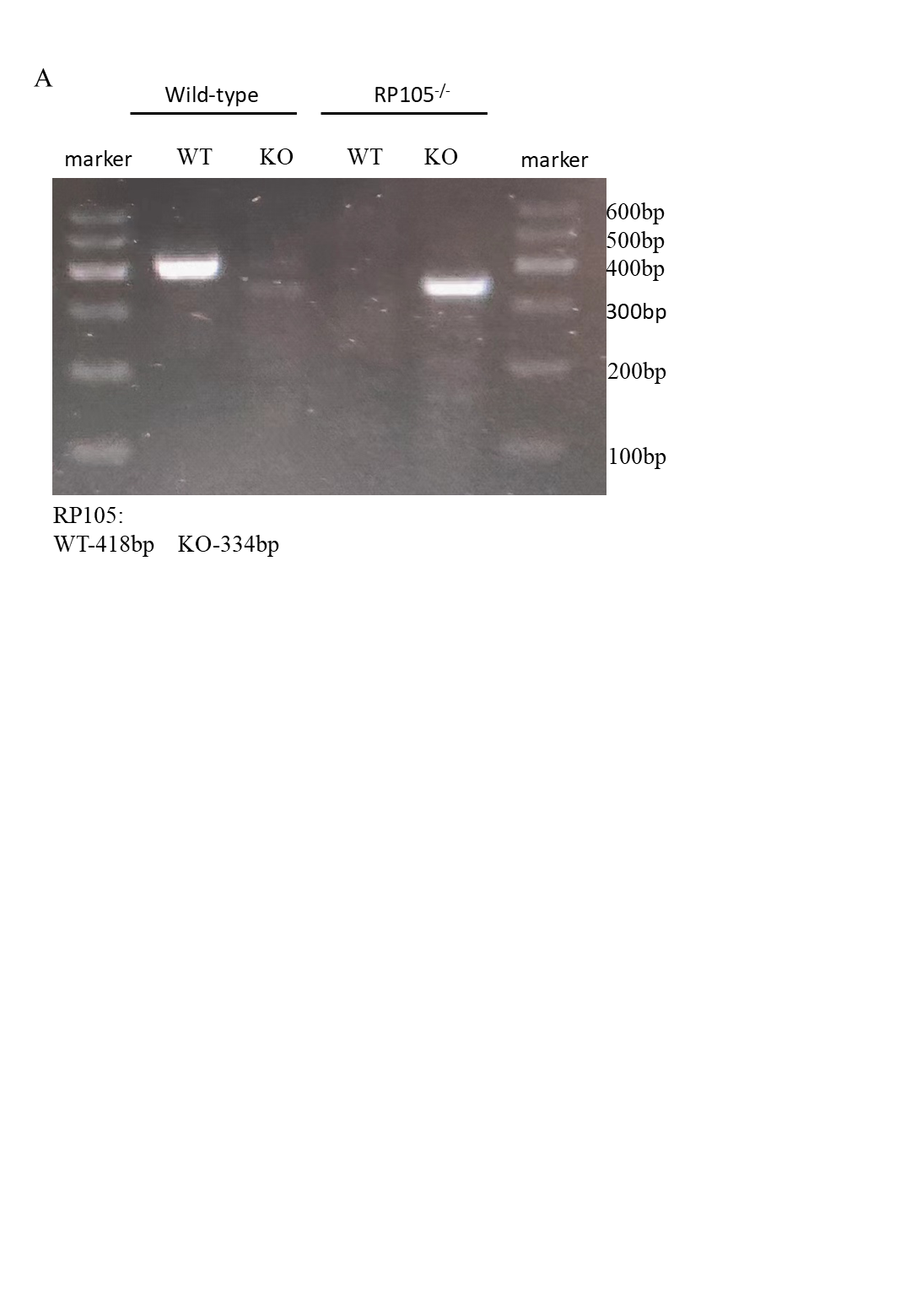

Supplement: Supplementary file 3 — Supplementary file, Fig. S2 [file 41420_2025_2578_MOESM3_ESM.tif]

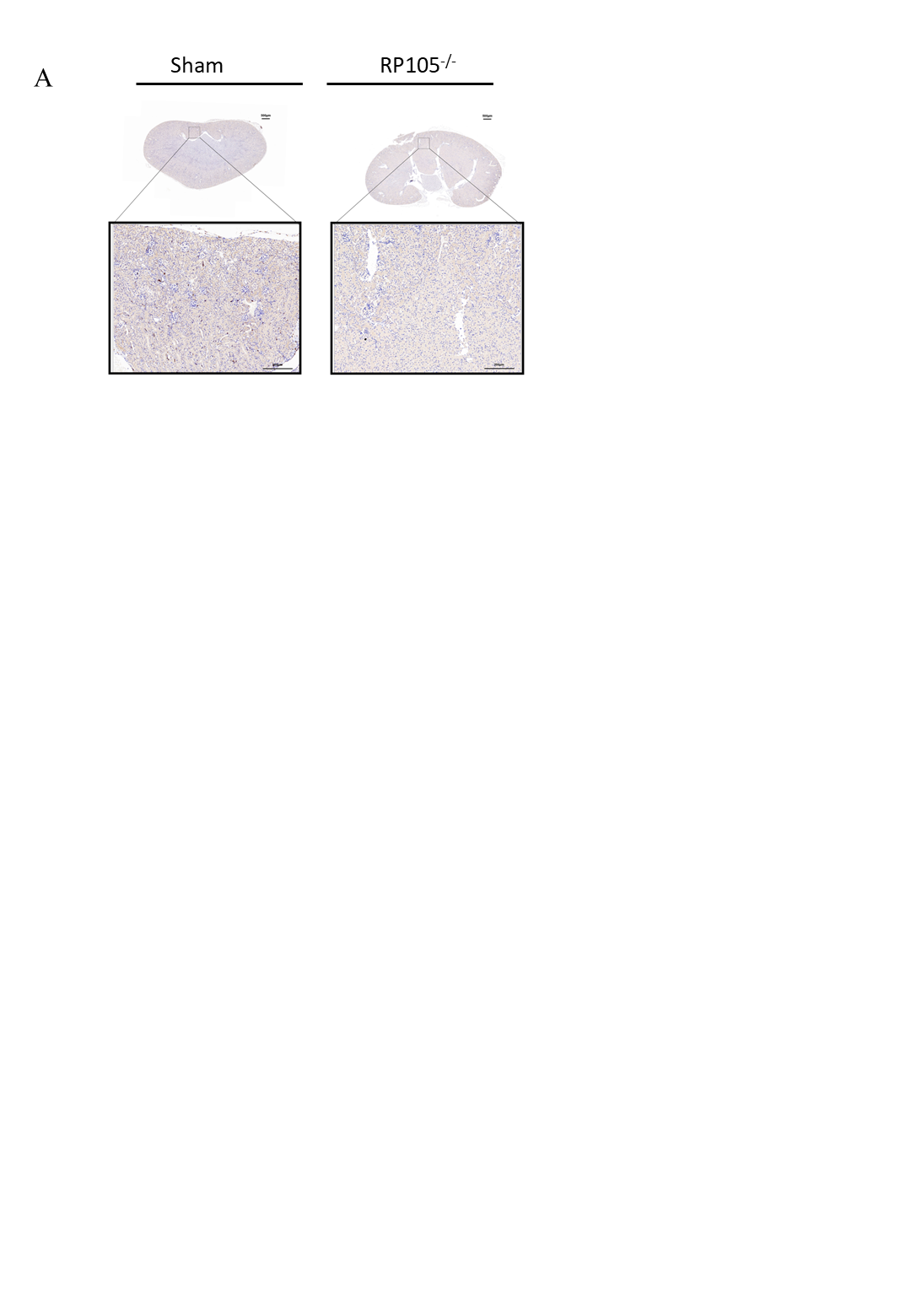

Supplement: Supplementary file 4 — Supplementary file, Fig. S3 [file 41420_2025_2578_MOESM4_ESM.tif]

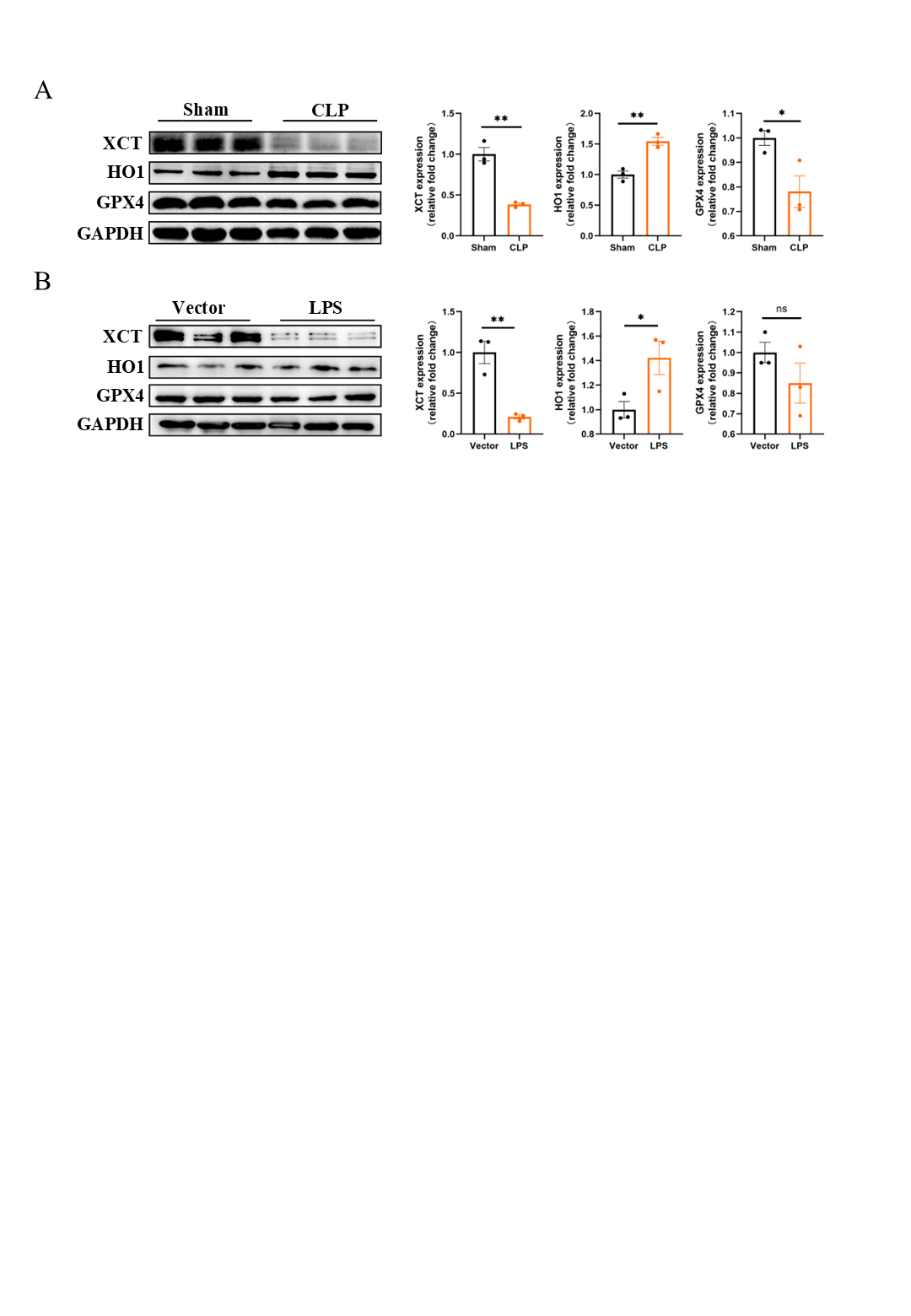

Supplement: Supplementary file 5 — Supplementary file, Fig. S4 [file 41420_2025_2578_MOESM5_ESM.tif]

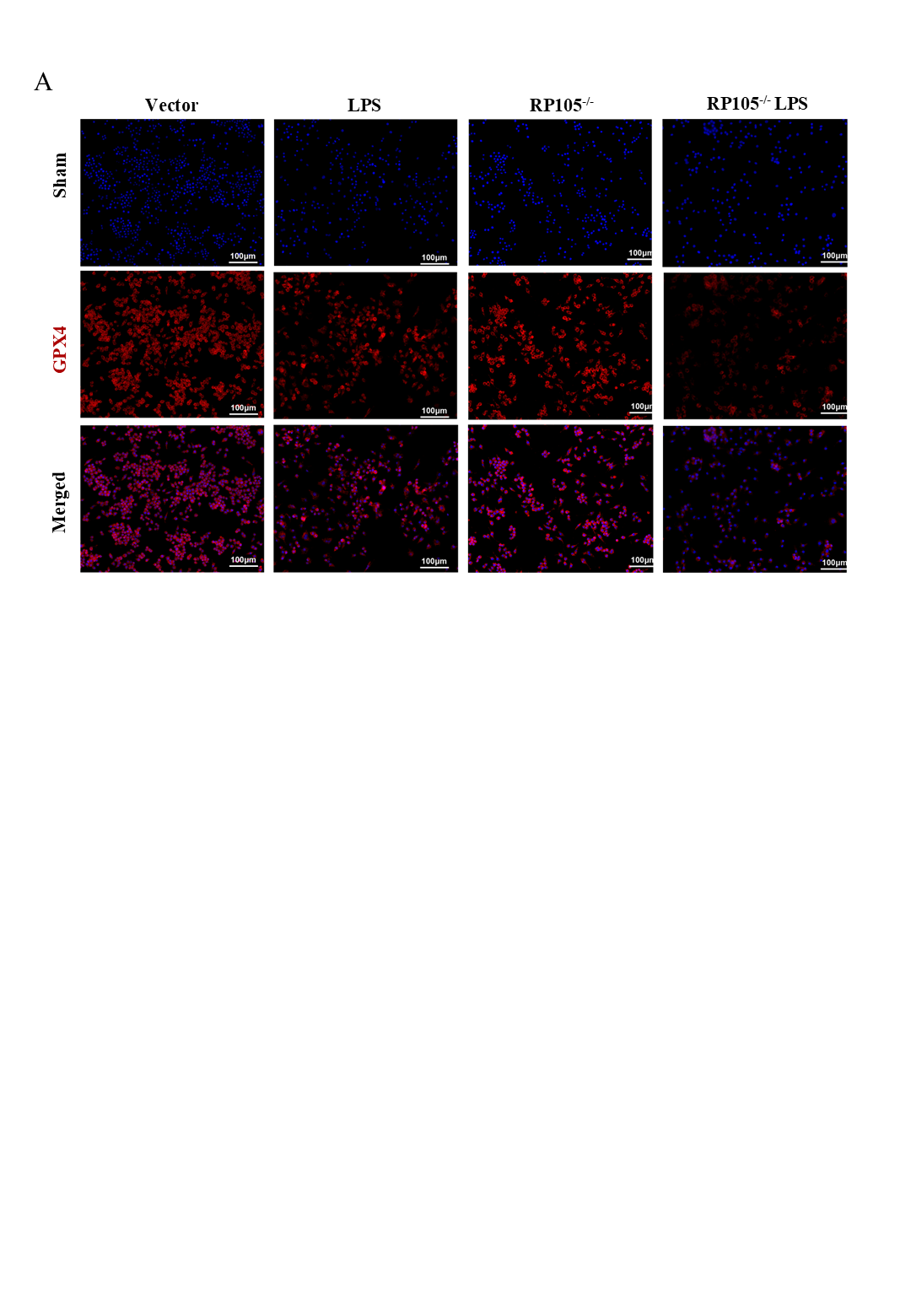

Supplement: Supplementary file 6 — Supplementary file, Fig. S5 [file 41420_2025_2578_MOESM6_ESM.tif]
