# Supplementary material for: Modulatory role of radioprotective 105 in mitigating oxidative stress and ferroptosis via the HO-1/SLC7A11/GPX4 axis in sepsis-mediated renal injury
Source: Cell Death Discov. 2025 Jul 1;11:290. doi: 10.1038/s41420-025-02578-7 (PMC12217763; doi:10.1038/s41420-025-02578-7)

Figure 1

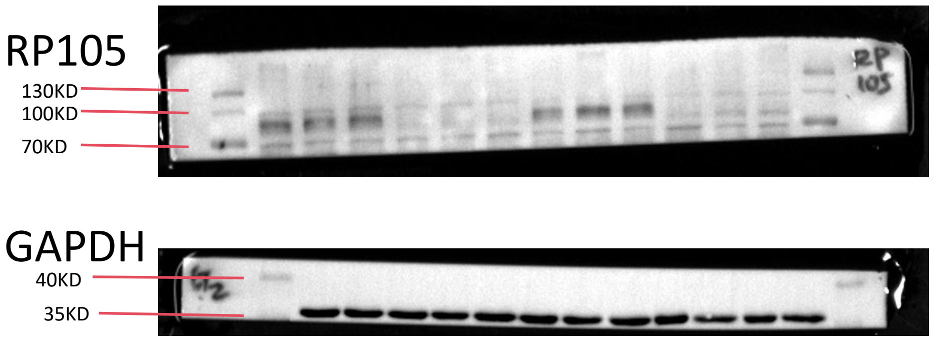

Figure 5

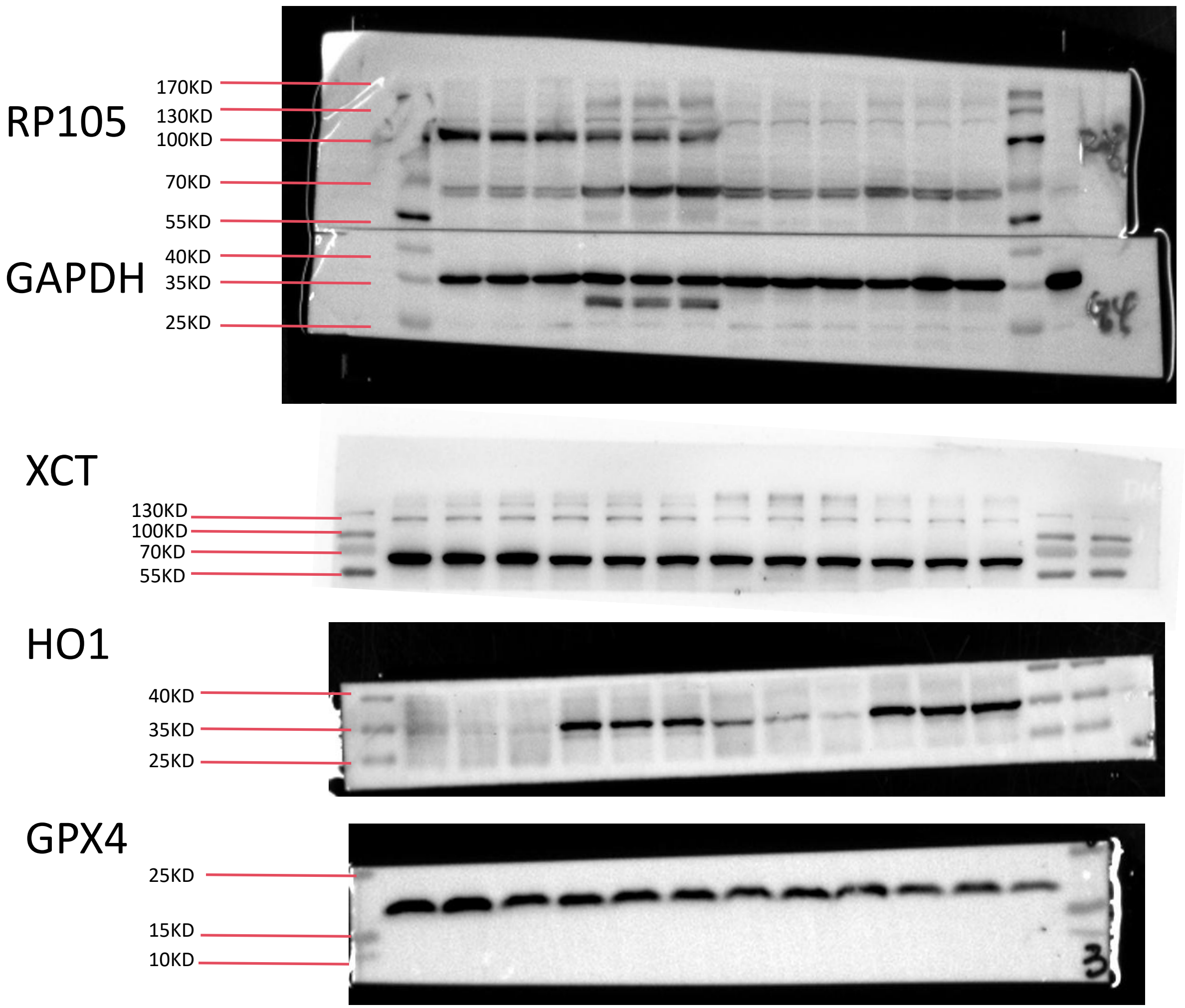

Figure 6

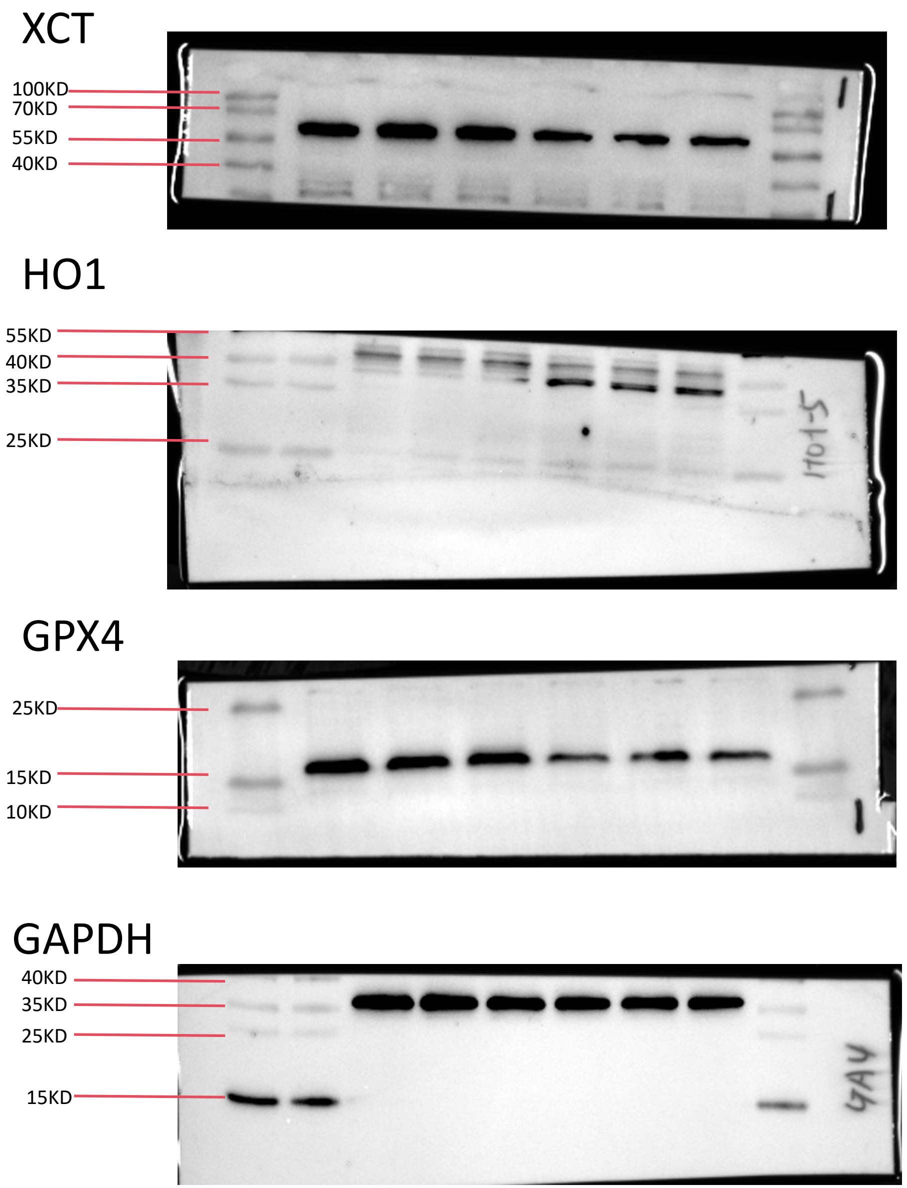

Figure 6

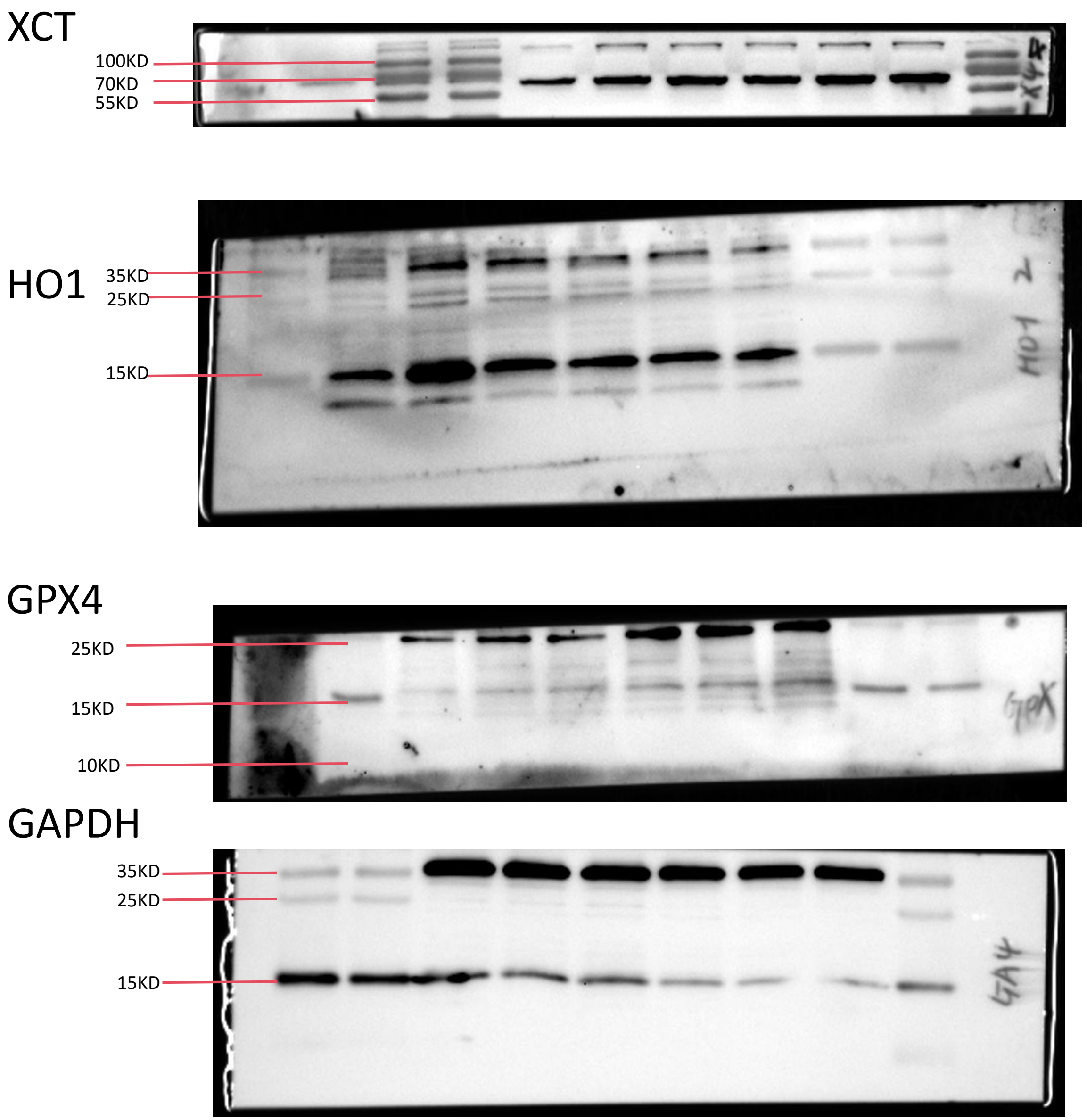

Figure 7

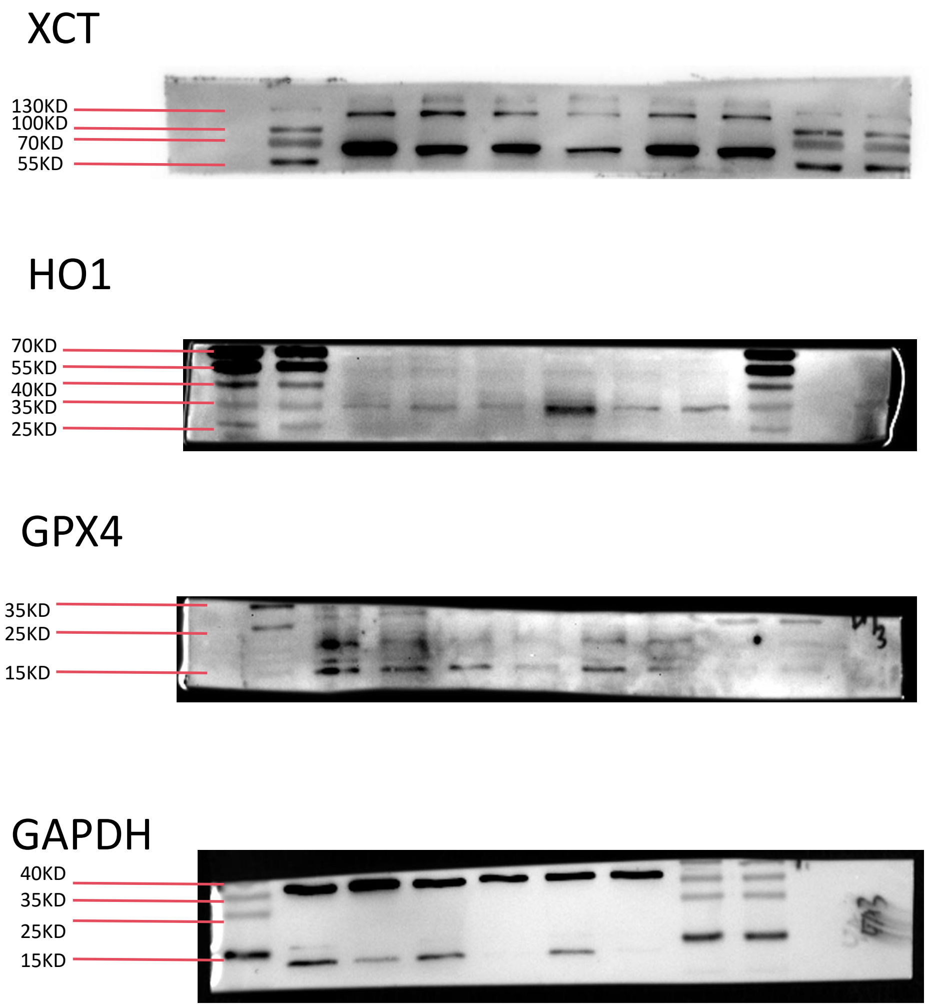
XCT
130KD
100KD
70KD
55KD
HO1
70KD
55KD
40KD
35KD
25KD
GPX4
35KD
25KD
15KD
GAPDH
40KD
35KD
25KD
15KD

Figure 8

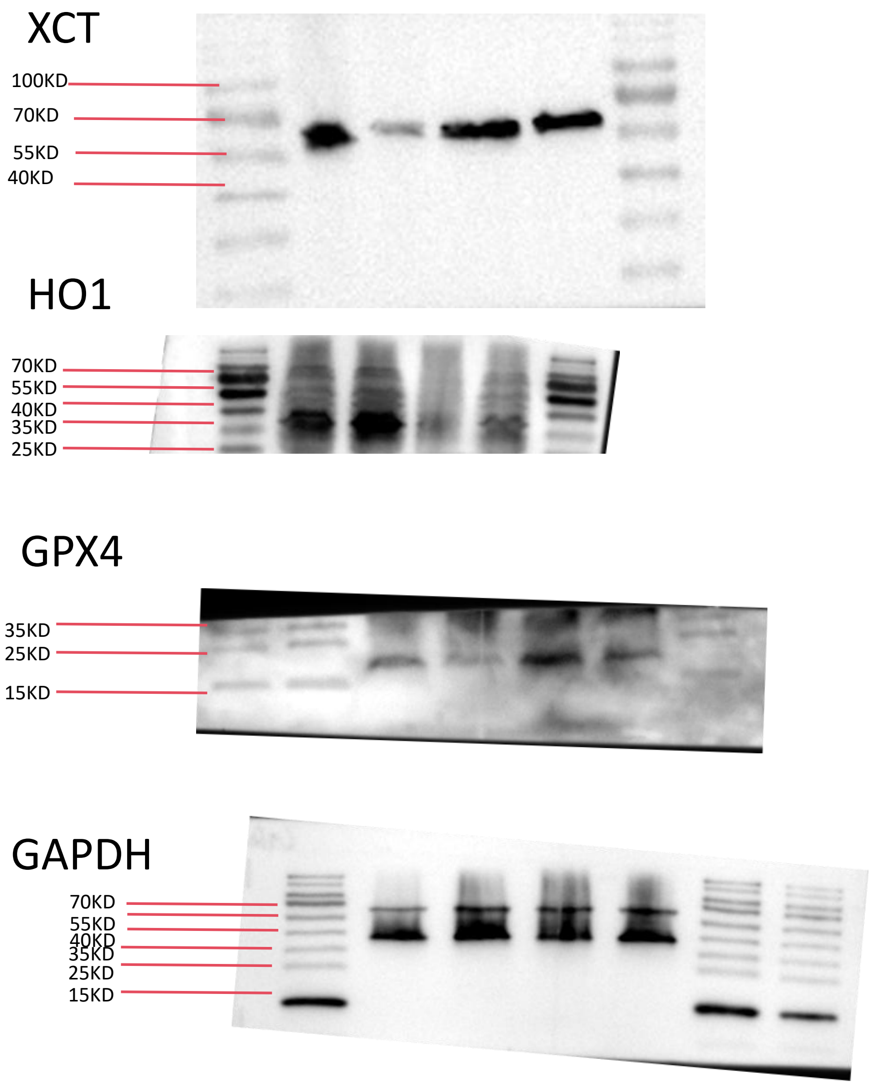

XCT
100KD
70KD
55KD
40KD
HO1
70KD
55KD
40KD
35KD
25KD
GPX4
35KD
25KD
15KD
GAPDH
70KD
55KD
40KD
35KD
25KD
15KD

Figure S4A

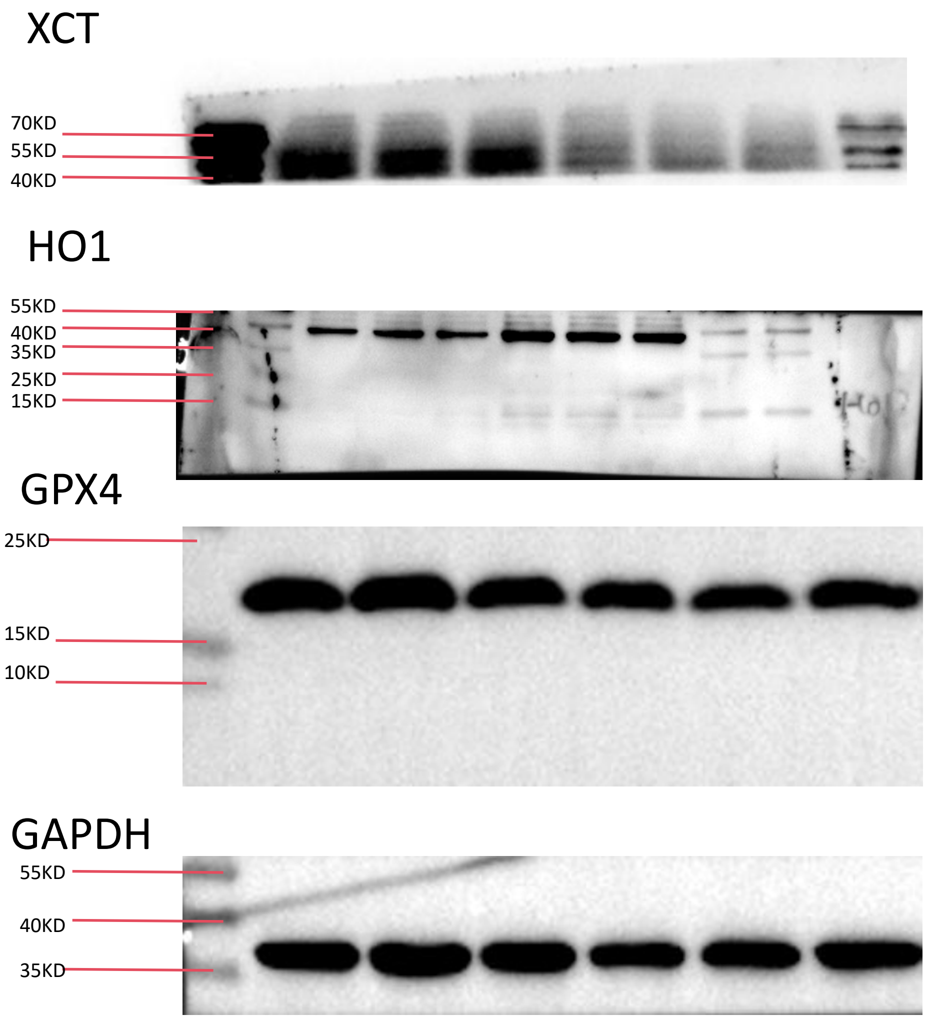

Figure S4B

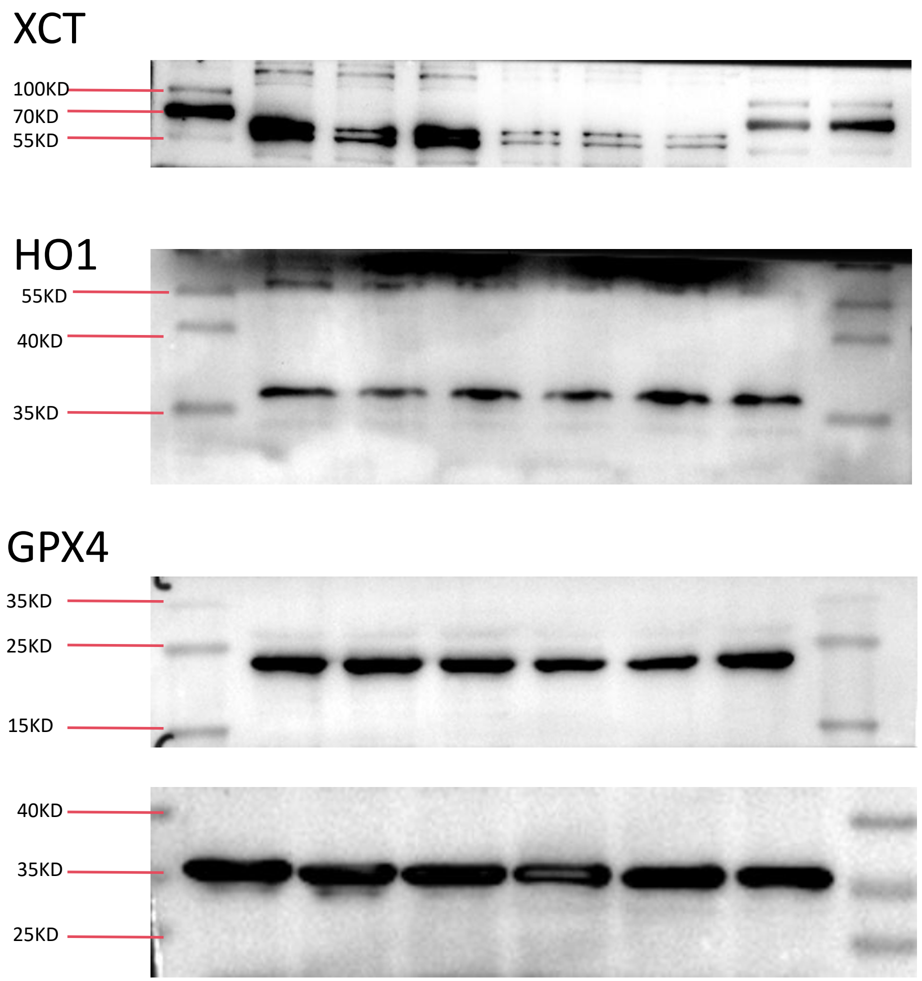

Supplement: Supplementary file 7 — Original Data [file 41420_2025_2578_MOESM7_ESM.pdf]
